# Swimming in the Air: The Power of Drag at Miniature Scales

**DOI:** 10.1093/icb/icag119

**Published:** 2026-07-20

**Authors:** Michael Sidebottom, Lee Margetts, Mostafa R A Nabawy

**Affiliations:** Department of Mechanical and Aerospace Engineering, School of Engineering, The University of Manchester, Manchester M13 9PL, UK; Department of Mechanical and Aerospace Engineering, School of Engineering, The University of Manchester, Manchester M13 9PL, UK; Department of Mechanical and Aerospace Engineering, School of Engineering, The University of Manchester, Manchester M13 9PL, UK; Aerospace Engineering Department, Faculty of Engineering, Cairo University, Giza 12613, Egypt

## Abstract

At the very edge of powered flight exist miniature insects with typical characteristic wing lengths of less than 1 mm. At such small scales, this domain of flight is characterized by Reynolds numbers ($Re$) of the order of 10 or less where the effects of air viscosity are significant. One distinguishable feature of miniature-scale flight is that of “drag-based” flapping kinematics, which are as closely related to the motions of swimming as they are to flying. The exact reason why miniature insects use drag-based kinematics, rather than the classical lift-based approach used by non-miniature insects, is not obvious. Hence, in this study, we investigate the above ambiguity via a quasi-steady aerodynamic approach. It is found that, for a stroke whose translational profile is symmetric about the half-period, pure-drag-based kinematics cannot be more aerodynamically effective or efficient than pure-lift-based kinematics. In fact, in almost all of the cases considered, lift-based flight is actually more advantageous than drag-based flight when half-strokes are symmetric. However, pure-drag-based kinematics become more favorable when we begin to consider flapping half-strokes that are asymmetric about the half period. When the asymmetry between the down- and up-stroke periods is increased, it is found that a force imbalance arises, via which viscous drag can be used to counteract the gravitational force. As such, the present study suggests that the reason why miniature insects do not use the conventional lift-based approach for flight, is a matter of taking advantage of their highly viscous environment, which allows for augmentation of both effectiveness and aerodynamic efficiency in hovering flight.

## Introduction

Miniature bristled winged insects are the smallest known flying creatures, with their scale leading to Reynolds numbers, $Re$, typically of the order of 10 or less. The uniqueness of this flight category can be seen in the swimming-like flapping profiles employed by miniature insects, which significantly depend on aerodynamic drag to counteract the gravitational force. For example, numerical assessments of hovering miniature insects have found that the contribution of drag to the time-averaged vertical force vector across a single stroke is 32% in the case of the *Paratuposa placentis* (Farisenkov et al. 2022), 50% in the case of thrips (Lyu et al. 2019), and 70% in the case of the *Encarsia formosa* (Cheng and Sun 2018). As such, their flight may be considered to be as much “drag-based” as it is “lift-based”. The reason why miniature insects have evolved so as to use the drag component to their advantage in a way that allows them to facilitate their extraordinary flight is unclear—some previous two-dimensional numerical studies have produced somewhat surprising results. Jones et al. (2015) studied a miniature-scale membranous aerofoil (i.e., one that is solid and without bristles) operating across a very low $Re$ range ($1\le Re \le 200$). They found that, while $Re \ge 3$, the stroke-averaged vertical force for a fixed flapping frequency, *f*, was greatest when performing a pure-lift-based stroke vs. a pure-drag-based profile. Whereas, when $Re < 3$, the average vertical force was marginally greater when considering drag-based kinematics vs. the lift-based kinematics. Shen et al. (2022) later carried out a similar study, this time for both membranous and bristled aerofoils, across the range $1\le Re \le 80$. For all $Re$ values they found that a membranous aerofoil would generate a greater stroke-averaged vertical force when performing a lift-based stroke, while also being slightly more aerodynamically efficient. Whereas, for the bristled aerofoil, the lift-based stroke was most effective and efficient only between $1\le Re \le 40$—for $40 < Re \le 80$, the drag-based stroke became increasingly more effective and efficient than the lift-based stroke. Although the findings from Jones et al. (2015) and Shen et al. (2022) differ in some places (mostly for $Re < 3$), they are both surprising in the same manner since they do not appear to coincide with the aforementioned observations of real miniature insects. If lift-based flight is more aerodynamically beneficial at $Re$ values pertinent to miniature insects, or, at least, if the difference between the two flight styles is somewhat negligible, then why are we yet to see a miniature insect using the classical lift-based approach that is by far the most conventional approach in every other flight domain? It should be highlighted that the two studies discussed above were performed using a Computational Fluid Dynamics (CFD) simulation of a two-dimensional wing. With that being said, the fact that we are not certain of the exact reason for the ambiguity encourages us to investigate the problem further and via an alternate method.

Obviously, miniature insects use neither the pure-lift- nor the pure-drag-based kinematics such as those assessed by Jones et al. (2015) and Shen et al. (2022). Instead, they tend to perform more complex wing motions which are somewhat a mixture of the two, and that are rather comparable to the motions of swimming. Mentioned earlier, was the study by Cheng and Sun (2018). In their investigation, the swimming-like flapping kinematics of the *E. formosa* were measured in order to numerically evaluate their aerodynamics—this included a comparison to those of a theoretical pure-lift-based profile. For $Re=10.6$, it was found that the time-averaged vertical force vector across a single stroke of flapping frequency 361 Hz would be greater for *E. formosa*’s swimming-like motion than for the lift-based profile by a factor of 3. While it might seem intuitive for the swimming-like profile to be far more effective at miniature scales (hence why miniature insects have evolved to use them), to conclude this based on the aforementioned study from Cheng and Sun (2018) is somewhat unfounded based on the authors’ model parameterizations: first, the swimming-like and lift-based profiles modeled in the study implicitly involve very different angular displacements throughout a single stroke cycle, whereby the overall angular displacement is greater for swimming-like profile than the lift-based profile. Hence, by implementing the same flapping frequency (i.e., 361 Hz) for both flapping styles, the authors render them somewhat incomparable since there is bound to be a disparity between the overall aerodynamic forces. Furthermore, the authors included an extra aerodynamic mechanism in their realistic simulation of the *E. formosa* known as the clap and fling mechanism, which did not feature in their lift-based assessment. The clap and fling mechanism refers to a wing–wing interaction which appears to be ubiquitous within many examples of miniature-scale flight and is known to notably increase circulation at a very early stage in the wing stroke (Lighthill 1973; Weis-Fogh 1975; Miller and Peskin 2005; Santhanakrishnan et al. 2018; Sidebottom et al. 2025a). The notable contribution made by the clap and fling mechanism to the overall vertical force can be seen in the data provided by Cheng and Sun (2018) during the fling motion, prior to the “forward sweep” portion of *E. formosa*’s flapping kinematics. During the fling, a noticeable force peak is generated reaching approximately 3.4$\times 10^{-7}$N (a factor of approximately 1.75 greater than the insect’s weight), which may further explain the disparity in their results, with regards to their comparison to the pure lift-based stroke.

The above critique is not intended to diminish the overall conclusion made by the authors, nor are we suggesting that swimming-like kinematics have no advantage at miniature scales—after all, the fact that we see such a technique ubiquitously in the miniature domain suggests that there is a phenomenon worth uncovering. Rather, the study from Cheng and Sun (2018) shows us that it is difficult to make any comparisons between lift- and drag-based kinematics, without firstly isolating them completely (i.e., without including additional aerodynamic mechanisms such as clap and fling) and then parameterizing for a fixed flight mode. A more satisfactory approach might include a quantification of the aerodynamic power required for generating a given aerodynamic load, which would allow for a more insightful comparison of the two kinematic styles. Such an approach was taken via numerical method by Engels et al. (2021) who compared a pure-lift-based profile to a figure-of-eight profile based on that of the *Nephanes titan* (Yavorskaya et al. 2019) under the conditions for hovering flight and without clap and fling. The two flapping styles were compared via the Rankine–Froude efficiency, which compares the calculated aerodynamic power to perform the stroke to that of an equivalent idealized rotor disk. Curiously, they did not discover a clear aerodynamic advantage to utilizing the swimming-like flapping profile over the lift-based profile. In fact, at wing-length-based $Re$ values of 4 and 57, lift-based kinematics were found to be the more aerodynamically efficient approach, and there was little difference between the two when $Re$ was 14. The exact reason why lift-based kinematics were found to be a more aerodynamically efficient flapping style at the highest and lowest tested $Re$ values yet not at the intermediate $Re$ value is not obvious and wasn’t assessed any further in the study. While the authors of the aforementioned computational study made some suggestions as to why we might see bristled wings at miniature scales, they made no attempt to explain the existence of swimming-like kinematics if the aerodynamics of lift-based styles are equally (if not more) efficient. Of course, the reason for the outlined ambiguity doesn’t have to be related to aerodynamics at all, and the reason why we see such peculiar flapping styles at miniature scales may be more related to biomechanical or physiological aspects of miniature insects, for instance. However, from the discussion provided here, it can be seen that the aerodynamics of miniature insects (particularly, when kinematics effects are concerned) remain a little ambiguous and that there is some disagreement as to whether drag-based flapping profiles have any aerodynamic-specific benefits at all over the classical lift-based approach.

**Fig. 1:**
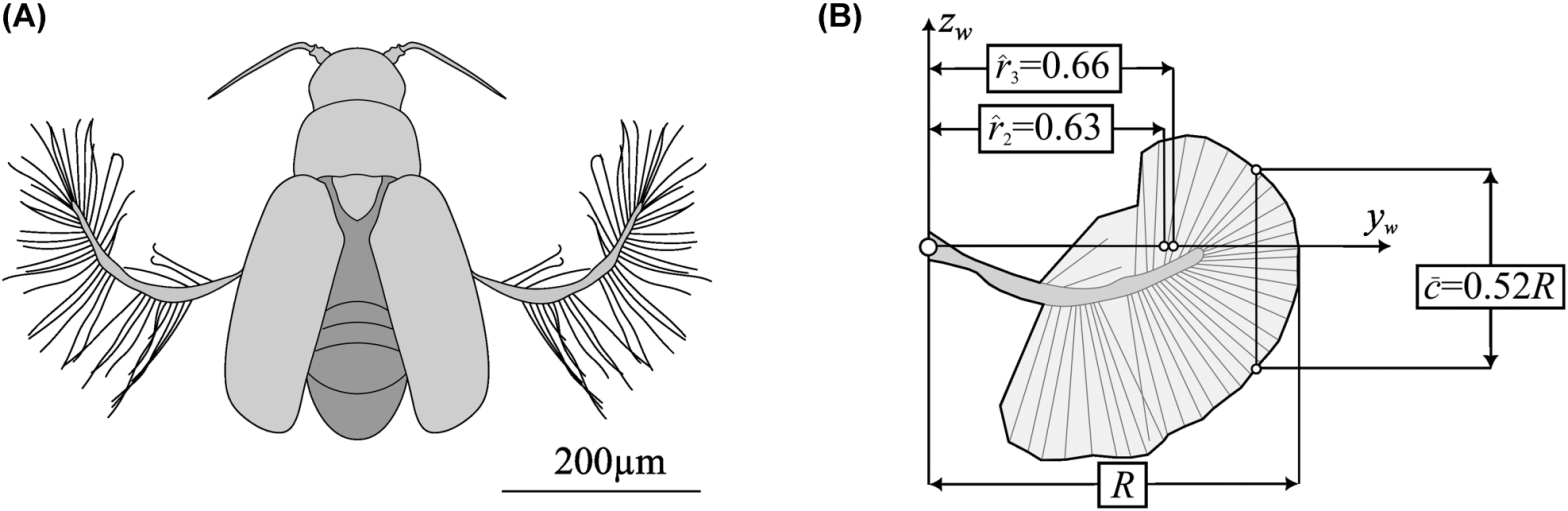
(A) Colloquially known as featherwinged beetles, Ptiliidae are a family of miniature insects whose wing morphologies are largely composed of long hair-like bristles extending from a central membrane. In the present study, we focus on a specific species of Ptiliidae called the *Paratuposa placentis*. (B) Geometrical representation of the *P. placentis’* wing as represented in the present study (see Table 1 for sources). Note that the annotated dimensions in (B) are based on a mechanical rigid wing model where wing/bristle flexibility was not considered.

In this paper, we investigate the above ambiguity via a semi-empirical quasi-steady approach. The quasi-steady approach allows for rapid analysis of insect aerodynamics and can provide useful insights in to the overall aerodynamic performance of a flapping insect wings, while avoiding the time expenses incurred by computational and experimental investigations (Ellington 1984a; Sane and Dickinson 2002; Berman and Wang 2007; Whitney and Wood 2010; Nabawy and Crowther 2014, 2015; Han et al. 2015; Wang et al. 2016; Nabawy 2023). Notably, the quasi-steady approach has yet to appear in analyses of flight in the miniature domain, likely due to the relatively small amount of empirical aerodynamic data available for miniature insect wings. However, owing to some studies on a species of featherwinged beetle (similar to that shown in Fig. 1a), such data is now available. In the present study, there are two key investigations. First, we compare the aerodynamic effectiveness (i.e., the magnitudes of the generated vertical aerodynamic forces) and efficiency (i.e., the aerodynamic power required to generate the force required for hovering flight) of *pure* lift- and drag-based kinematics while maintaining kinematic symmetry of half-strokes across a range of $Re$ values below 50. Our findings suggest that there is little difference between the lift- and drag-based profiles and that, for all-but-one parameter setting, lift-based kinematics in fact outperforms drag-based kinematics. Hence, in the second analysis, we consider kinematic asymmetry, whereby the total stroke period is maintained and the duration of the individual half-strokes are proportionally lengthened and shortened. Stroke asymmetry has been very briefly investigated in one of the aforementioned two-dimensional CFD studies [i.e., Jones et al. (2015)], in which it was found that the vertical force vector can be augmented for the drag-based stroke by increasing the velocity of the downstroke with respect to the upstroke. However, the exact reason why this was found to be the case was not explored in great depth by the authors, and no comparison to an asymmetric lift-based stroke was provided. Nonetheless, they attributed their findings for the drag-based stroke to the generation of larger vortices during the downstroke than the upstroke for larger stroke asymmetries and that this phenomena is more pronounced for higher $Re$ approaching 100. Whether or not it is true that the production of larger vortices on one half-stroke will lead to greater aerodynamic loads across that half-stroke, this effect is hardly confined to the miniature domain—which the authors point out themselves—and isn’t necessarily specific to drag-based kinematics either. Therefore, in this study, we use the quasi-steady assumption to provide an alternate explanation to the above phenomena, revealing that the skin friction component creates an aerodynamic advantage to using drag-based kinematics over lift-based kinematics at miniature scales when employing asymmetric half-strokes. This effect is most pronounced at miniature scales and therefore our explanation makes sense even within the general context of flapping wing flight.

## Model

### Geometric and kinematic parameters

Across two experimental studies (Kolomenskiy et al. 2020; Farisenkov et al. 2022), the geometric, kinematic, and aerodynamic characteristics of a species of miniature featherwing beetle (Order: *Coleoptera*; Family: *Ptiliidae*; Species: *P. placentis*, see Fig. 1A) have been defined. The geometric parameters relevant for this study include the wing length, *R*, the mean geometric chord, $\bar{c}$, and the non-dimensional radius of second and third moments of wing area, $\hat{r}_2$ and $\hat{r}_3$, respectively (see Fig. 1B and Table 1). The relevant kinematic parameters include the flapping frequency, *f*, and the maximum flapping angle, $\phi _{amp}$. The insect’s mass, *m*, was also useful for this investigation, in order to indicate whether a particular hovering kinematic profile could enable weight balance. Note that $\hat{r}_{3}$ was calculated using a statistical expression defined by Ellington (1984b) for the non-dimensional radius of third moment of wing area ($\hat{r}_{3}= (0.954(\hat{r}_{2})^{0.794}$).

**Table 1:** Geometric, kinematic, and inertial parameters used within the current model

Parameter	Symbol	Value	Reference/comment
body mass (*μ*g)	*m*	2.86	Farisenkov et al. (2022)
wing length (mm)	*R*	0.495	Farisenkov et al. (2022)
mean chord (mm)	c̄	0.52*R*	Kolomenskiy et al. (2020)
radius of second moment of wing area (mm)	$\hat{r}_{2}$	0.63	Kolomenskiy et al. (2020)
radius of third moment of wing area (mm)	$\hat{r}_{3}$	0.66	Ellington (1984b)
flapping frequency (Hz)	*f*	184	Farisenkov et al. (2022)
maximum flapping angle (deg.)	$\phi _{amp}$	90	Farisenkov et al. (2022)

*Note*: The majority of the parameters were extracted directly from one of two studies on the *P. placentis*, whereas $\hat{r}_3$ was calculated using an expression defined in the referred paper, where $\hat{r}_3$ is defined as a function of $\hat{r}_2$.

### Motion representation

**Fig. 2:**
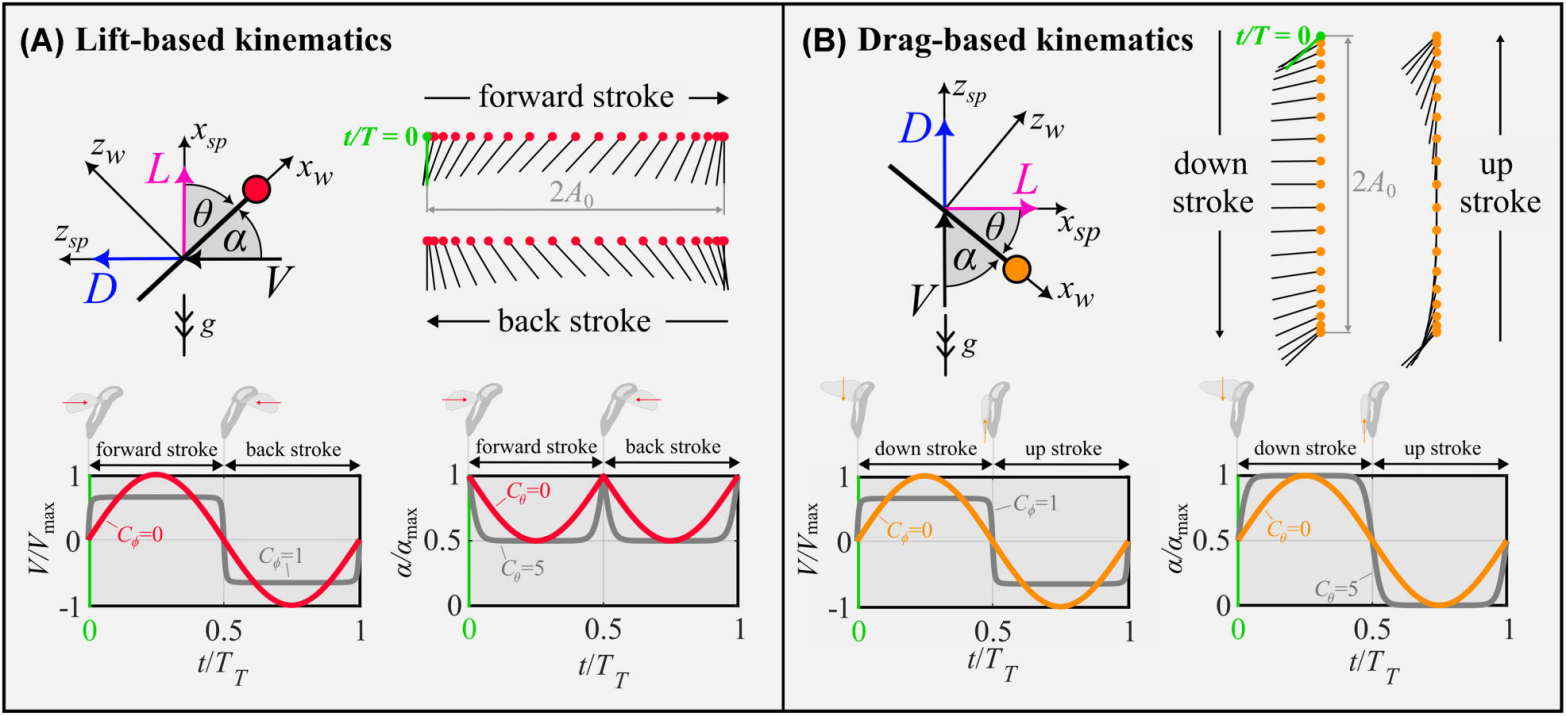
Symmetric (A) lift- and (B) drag-based kinematics. The stick diagrams illustrate the wing chord’s position at 42 evenly spaced time increments across a full stroke where the green sticks indicate the wing’s position at $t/T=0$ and the distance travelled by the wing across a half-stroke is $2A_0$. The two-dimensional reference frames are those of the stroke-plane-fixed frame ($x_{sp}, z_{sp}$) and the wing-fixed frame ($x_{w}, z_w$), where the pitching angle, $\theta$, refers the angle between $x_{sp}$ and $x_w$, whereas the wing’s geometric angle of attack, $\alpha$, refers to the angle between $x_w$ and the translational velocity vector, *V*. Throughout an entire stroke of the lift-based kinematics, the $z_{sp}$ axis (and therefore the stroke plane) is fixed perpendicular to the gravitational force vector, *g*, whereas, for the drag-based stroke, it is fixed parallel with *g*. Furthermore, for both flapping styles, *V* aligns with the $z_{sp}$ axis throughout an entire stroke. Hence, the only force component which counteracts the gravitational force is that of *L* and *D* for the lift- and drag-based strokes, respectively. The waveforms outline *V* and $\alpha$ with respect to non-dimensional time ($t/T$) across a full stroke, where *V* and $\alpha$ have been normalized with respect to their maximum values, $V_{\mathrm{max}}$ and $\alpha _{\mathrm{max}}$, respectively. $V_{\mathrm{max}}$ and $\alpha _{\mathrm{max}}$ occur during sinusoidal translation and pitching variations represented by the red and orange curves and corresponding to $C_\phi$ and $C_{\theta }$ values of 0. The gray curves represent rectangular and near-rectangular variations in *V* and $\alpha$, respectively, resulting from $C_\phi$ and $C_{\theta }$ values of 1 and 5, respectively. Note that the condition $C_{\phi } = 1$ leads to a lower $V_{\mathrm{max}}$ when compared to sinusoidal condition of $C_{\phi }=0$.

In this study, we only consider the effects of pure lift- or pure drag-based kinematics in hovering flight. This implies that the kinematic wing profiles must be ones which are either aligned with the global horizontal plane or the global vertical plane, respectively (i.e., perpendicular or parallel to the gravitational force vector, see Fig. 2). This decision is necessary to ensure an objective comparison between the two extreme cases, where the gravitational force is counteracted by only one of the two aerodynamic force components. While real miniature insects will most-likely be adopting a mixture of the two motion profiles, our aim is not to simulate real wing motions but rather to establish an understanding of the merits of each case. As such, both the lift- and drag-based profiles only require two-degrees-of-freedom: translational and pitching rotation.

To explain why the flapping motion has been defined as a translational degree of freedom, rather than a rotational one, this is because the kinematic motions assessed in this investigation use only one of the two aerodynamic force components (i.e., lift or drag) to counteract the gravitational force. However, if rotational flapping kinematics were to be considered for the drag-based stroke, then this counteraction would be effected by both the drag *and* the lift component throughout the majority of the wing stroke. Hence, to avoid this, the flapping waveform is defined such that the displacement of the wing is translational rather than angular. Therefore, throughout this paper we refer to the wing’s “translational” motion rather than its “flapping” motion. In this manner, during the lift-based stroke, the wing’s velocity vector will be continuously *perpendicular* to the global vertical plane, whereas, during the drag-based stroke, the wing’s velocity vector will be continuously *parallel* to the global vertical plane.

Before outlining the mathematical expressions for the wing motion waveforms, it is necessary to first explain how and why stroke asymmetry, denoted by $\sigma$, has been considered and integrated in to the kinematic framework. In nature, we see miniature insects performing different portions of their strokes at different velocities. For instance, within the figure-of-eight-shaped profile performed by the *P. placentis*, the so called “power” and “recovery” strokes have been observed, describing rapid downward motions and slower upward motions, respectively (Farisenkov et al. 2022). Similarly, the *E. formosa* uses “rowing” and “sweeping” motions in which most of the overall vertical force is generated during the faster rowing portion, while the sweeping portion is slower and returns the wing back to the starting position of the rowing portion (Cheng and Sun 2018). Conversely, however, the contrary to the above has been observed in a couple of other studies, which were collated by Jones et al. (2015), in which the upstroke was found to be performed at a larger velocity than the downstroke in the *E. formosa* (Weis-Fogh 1973) and also Thrips (Santhanakrishnan et al. 2014). To simulate either case, we have introduced $\sigma$, which controls the difference between the duration of the first half-stroke, $T_1$, and that of the second half-stroke, $T_2$, via the following relationship:


(1) \begin{eqnarray*}
T_1 + T_2 = \frac{1}{f}
\end{eqnarray*}

across the range $-1< \sigma < 1$. $T_1$ is defined as a function of *f* and $\sigma$ via:


(2) \begin{eqnarray*}
T_1 = \frac{(1+\sigma )}{2f}.
\end{eqnarray*}

It is useful to point out that Equation 2 is defined so that $\sigma =0$ corresponds to a symmetrical stroke (i.e., $T_1 = T_2$) and a negative or positive value of $\sigma$ simply implies a shortening of the first or second half-stroke, respectively. It was deemed necessary to investigate both positive and negative values of $\sigma$ in this study, since those studies outlined above, in which real-time recordings of flying miniature insects have been taken, seem to present opposing findings.

The mathematical representations of the kinematic waveforms prescribed in this study are based upon those in (Nabawy and Crowther 2015; Berman and Wang 2007) with an adjustment to accommodate for stroke asymmetry. As such, the translational wing velocity, *V*, is defined by


**Fig. 3:**
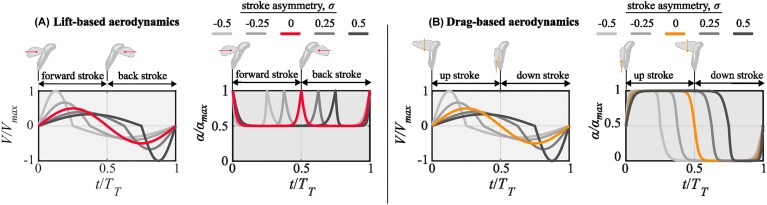
Translational velocity (*V*) and geometric angle of attack ($\alpha$) waveforms for (A) lift-based kinematics and (B) drag-based kinematics, for variations in $\sigma$, normalized with respect to the maximum of each throughout the stroke cycle. The waveforms correspond to $C_{\phi }=0$ (which is the “realistic” flapping condition used in this assessment), $C_{\theta }=5$ (which is the pitching conditions required for maximizing both effectiveness and efficiency for symmetric half-strokes, outlined in Section “Aerodynamic performance for symmetric profiles”) and $\theta _{amp}=45^\circ$ (which is the condition for maximizing effectiveness for symmetric half-strokes, also outlined in Section “Aerodynamic performance for symmetric profiles”). We have selected to display the *V* variations in this figure in order to show how $\sigma$ notably influences the maximum translational velocity performed throughout the stroke. Note that, for $V/V_{\mathrm{max}}$ plots, the translational velocity variations are identical for both lift- and drag-based kinematics and are defined according to Equation 3, whereas the angle of attack variations are different for both flapping styles, and change as according to Equation 8. However, the value of $\alpha _{\mathrm{max}}$ is the same in each plot (i.e., $90^\circ$).

(3) \begin{eqnarray*}
&&V(t)=\\&&\left\{\begin{array}{cc}A_0 \cdot \frac{\pi\left[t+t_0\right]}{T_n} \cdot \sin \left(\frac{\pi\left[t+t_0\right]}{T_n}\right) & C_\phi=0 \\-\frac{A_0}{\sin ^{-1} C_\phi} \cdot \frac{\pi C_\phi}{T_n} \cdot \frac{\sin \left(\frac{\pi\left[t+t_0\right]}{T_n}\right)}{\sqrt{1-C_\phi^2 \cos \left(\frac{\pi\left[t+t_0\right]}{T_n}\right)^2}} &\,\,\, 0< C_\phi \leq 1\end{array}\right.,\\
\end{eqnarray*}

where $C_{\phi }$ controls the shape of variation of the translational profile, whereby $C_{\phi }=0$ corresponds to a sinusoidal variation in translational velocity whereas $C_{\phi }=1$ corresponds to a constant translational velocity (illustrated in Fig. 3). $A_0$ is a constant that is linked to the amplitude of the waveform (Nabawy and Crowther 2015), and since we are considering a translational motion rather than rotational, the constant is defined by


(4) \begin{eqnarray*}
A_0 = (R\hat{r}_k)\phi _{\mathrm{amp}},
\end{eqnarray*}

where $\hat{r}_k$ is $\hat{r}_2$ for any force calculation and is $\hat{r}_3$ for any aerodynamic power calculation, as was implemented by Li and Nabawy (2022a, 2022b). Because for a hovering wing, the velocity distribution will vary along the wing span, then the correct reference velocity to use is that experienced at $\hat{r}_2$ for lift and drag calculations and at $\hat{r}_3$ for aerodynamic power calculations (Ellington 1984b).

**Fig. 4:**
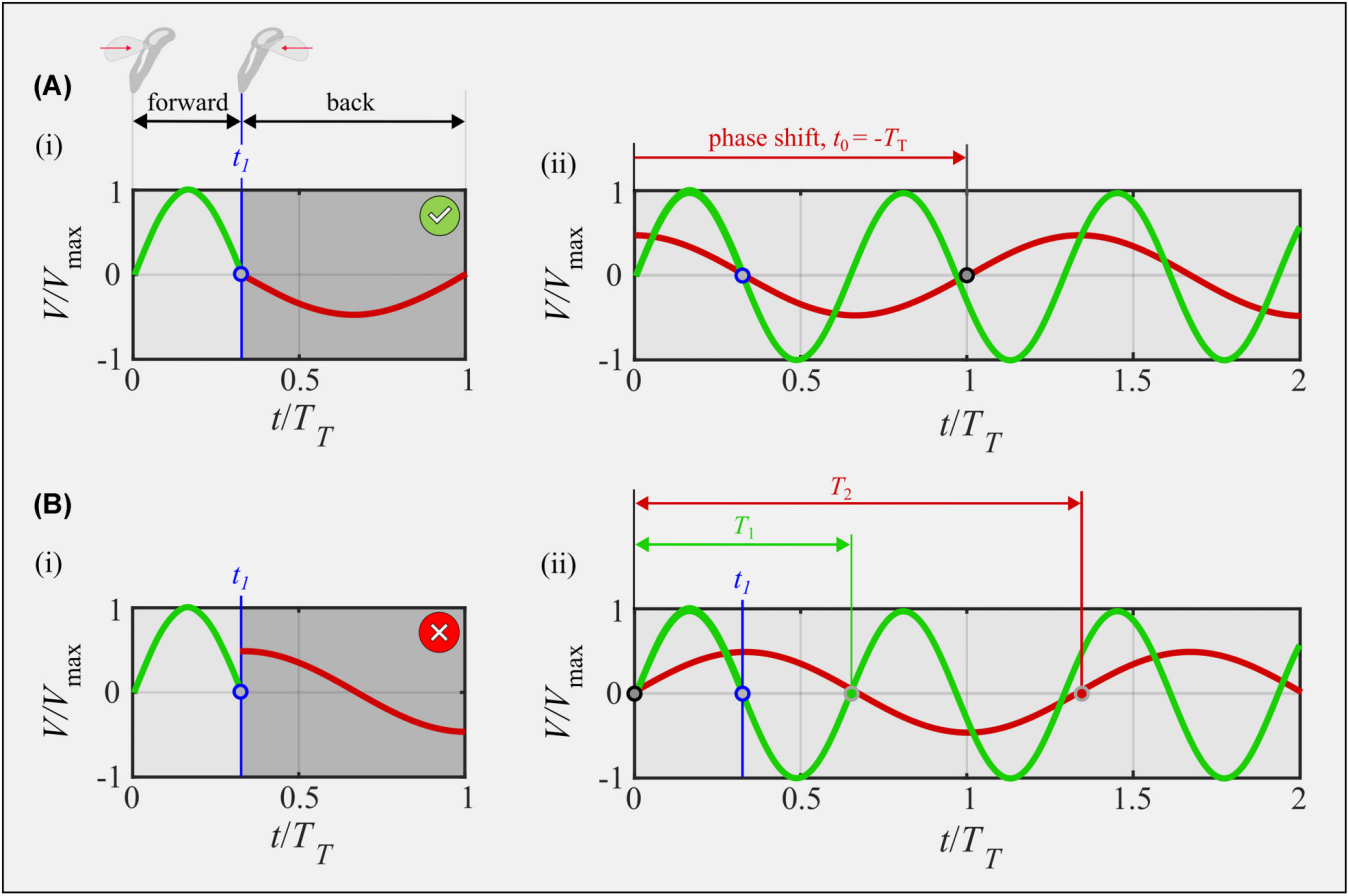
(A) (i) An exemplar asymmetric velocity waveform corresponding to $\sigma = 0.35$ plotted against non-dimensional time, $t/T_T$, for a full stroke. (ii) It can be seen that the full waveform actually consists of two separate waveforms (i.e., green and red for the forward and back strokes, respectively) with two different time periods, $T_1$ and $T_2$, respectively. However, for the green waveform to correctly transition to the red waveform at $t_1$ (i.e., the blue marker at $t_1 = T_1/2$) a phase shift, $t_0$, of $-T_T$ is required for the red waveform. In keeping with the nature of stroke reversal, this phase-shift is necessary so that the transition occurs at $V = 0$ ms$^{-1}$ and the sign of *V* becomes negative to indicate the change in wing direction. Note that the waveforms in (ii) are plotted across $0 \le t/T_T \le 2$ for demonstration purposes only, in order to reveal the full period of the red waveform, $T_2$. Conversely, in (B), (i) the representative waveform which occurs when a phase shift isn’t applied to the red waveform [as shown in (ii)] is illustrated so that *V* instantaneously jumps to a positive non-zero value at the beginning of the back stroke. Note that $t_0 = -T_T$ is correct for all values of $\sigma$.

In order to capture the change in velocity waveform at $t=t_1$ at the end of the first half-stroke (see Fig. 4), $T_n$ is defined by


(5) \begin{eqnarray*}
T_n = \left\lbrace \begin{array}{@{}l@{\quad }l@{}}\begin{aligned} &T_1 \:\:\:\:\:\:\:\: 0 \le t \le t_1 \\&T_2 \:\:\:\:\:\:\: t_1 < t \le T_T \end{aligned} \end{array}\right.,
\end{eqnarray*}

where $T_T$ is the period of the full stroke (i.e., $T_T = 1/f$). Then, $t_0$ is a time phase shift which ensures a smooth transition from the first half-stroke to the second for any value of $\sigma$ and is defined by


(6) \begin{eqnarray*}
t_0 = \left\lbrace \begin{array}{@{}l@{\quad }l@{}}\begin{aligned} &\:\:\:\:\ 0 \:\:\:\:\:\:\:\:\:\:\:\: 0 \le t \le t_1 \\& -T_T \:\:\:\:\:\:\:\:\: t_1 < t \le T_T \end{aligned} \end{array}\right..
\end{eqnarray*}

The reader is referred to Fig. 4 where the definitions of $T_n$ and $t_0$ are further explained to highlight their importance for correct modeling of the asymmetric kinematics. Next, the pitching angle time variation is defined by


**Fig. 5:**
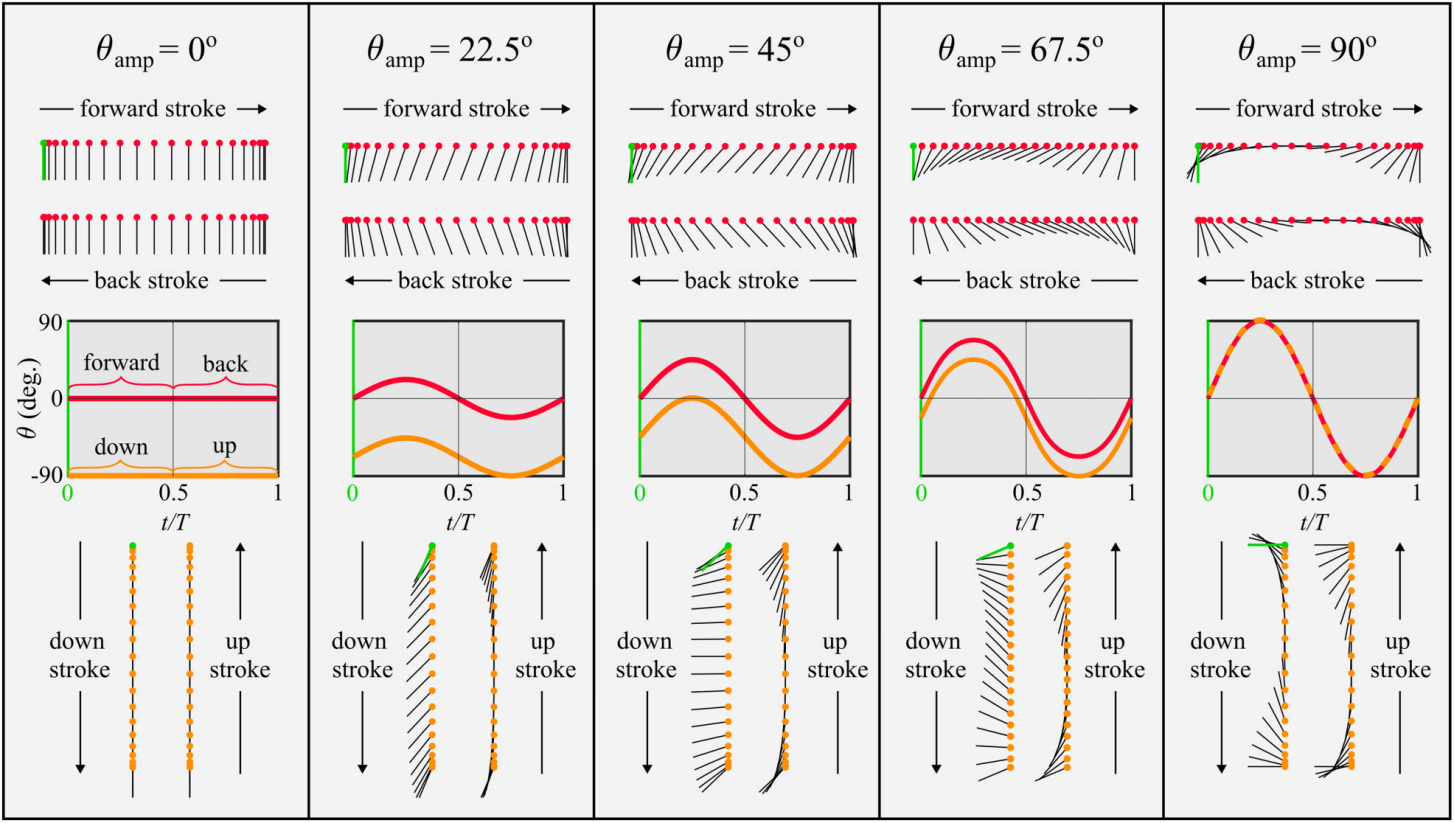
Lift-based (red) vs. drag-based (orange) pitching kinematics for various $\theta _{amp}$ settings. During the pure drag-based stroke, $\theta$ always reaches a value of $90^\circ$ during the upstroke, owing to the definition of $\beta$ (see Equation 7) which ensures that the wing returns to $\alpha = 0^{\circ }$ at the mid-point of the upstroke. The definition of $\beta$ implies that the pitching variations in the lift- and drag-based strokes coincide when $\theta _{amp}=90^\circ$. The reader should note that the shapes of the pitching waveforms change according to $C_\theta$ and here we have only illustrated the case of $C_{\theta }=0$ purely to avoid overcrowding in the figure.

(7) \begin{eqnarray*}
&&\theta(t)=\\&&\left\{\begin{array}{cc}\theta_{\mathrm{amp}} \cdot \sin \left(\frac{\pi\left[t+t_0\right]}{T_n}\right)+\beta & C_\theta=0 \\\frac{\theta_{\mathrm{amp}}}{\tanh C_\phi} \cdot\left(\tanh \left[C_\theta \sin \left(\frac{\pi\left[t+t_0\right]}{T_n}\right)\right]\right)+\beta &\,\,\, 0< C_\theta< \infty\end{array}\right.,\\
\end{eqnarray*}

where $C_{\theta }$ controls the shape of variation of the pitching profile, whereby $C_{\theta } = 0$ corresponds to a sinusoidal pitching variation and $C_{\theta }\rightarrow \infty$ approaches a rectangular pitching variation (hence a constant angle of attack). $\beta$ is a translation of the pitching waveform which offsets the pitching function by a constant value—in the present work, $\beta$ takes the value of zero and $(\pi /2-\theta _{amp})$ for the pure lift- and pure drag-based kinematics, respectively. The inclusion of $\beta$ ensures that the angle of attack of the wing will always be zero at the mid-upstroke of drag-based kinematics (see Fig. 5), which is the desirable wing position for minimizing drag during the upstroke in order to maximize the overall net upward force across the full stroke. Finally, the time variation of the geometric angle of attack (which is visually shown in Fig. 3) can be written as


(8) \begin{eqnarray*}
\alpha (t) = \left\lbrace \begin{array}{@{}l@{\quad }l@{}}\begin{aligned} & \:\ \frac{\pi }{2}- |\theta (t)|, \:\:\text{lift-based kinematics}\\& \:\ |\theta (t)-\frac{\pi }{2}|, \:\:\text{drag-based kinematics} \end{aligned} \end{array}\right..
\end{eqnarray*}

### Modeling assumptions

The primary aerodynamic mechanisms relating to flapping insect wings are fourfold and include wing translation, wake-capture, wing rotation, and the added-mass effect (Li et al. 2025). Generally, wing translation provides the most significant contribution to the overall aerodynamic coefficients, owing to the creation of a stable leading-edge vortex (LEV) and the subsequent absence of stall at high angles of incidence. We outline the relevant translatory coefficients in the subsequent subsection. With regards to wake-capture, recent Particle Image Velocimetry (PIV) flow structure analysis suggests that its effect on aerodynamic performance at miniature scales may be considerably diminished owing to heightened viscous flow effects (O’Callaghan and Lehmann 2023). In fact, this is consistent with a recent analysis that suggests that wake capture effects become negligible at low $Re$ (Nabawy 2023), hence the expectation is that they will be significantly reduced for the scale of $Re$ of concern here and can be safely neglected. Furthermore, because there is no advance/delay in pitch with respect to flapping for the present analysis, the *net* effects of wing rotation and added mass become negligible (since the effects of both mechanisms will always be reversed). Moreover, we assume the system to be perfectly elastic, meaning that the power required to overcome inertial effects is fully compensated by the elastic energy stored (Phan and Park 2018). Therefore, assuming perfect elasticity means that the only contributor to the overall mechanical power required by the insect for flight is that of aerodynamic power. This is of course an idealization of the reality of flapping wing aerodynamics, and we do not overlook that this is a limitation of the present study. However, the purpose of this investigation is to focus only on the aerodynamics, justifying this decision.

### Aerodynamic coefficients

**Fig. 6:**
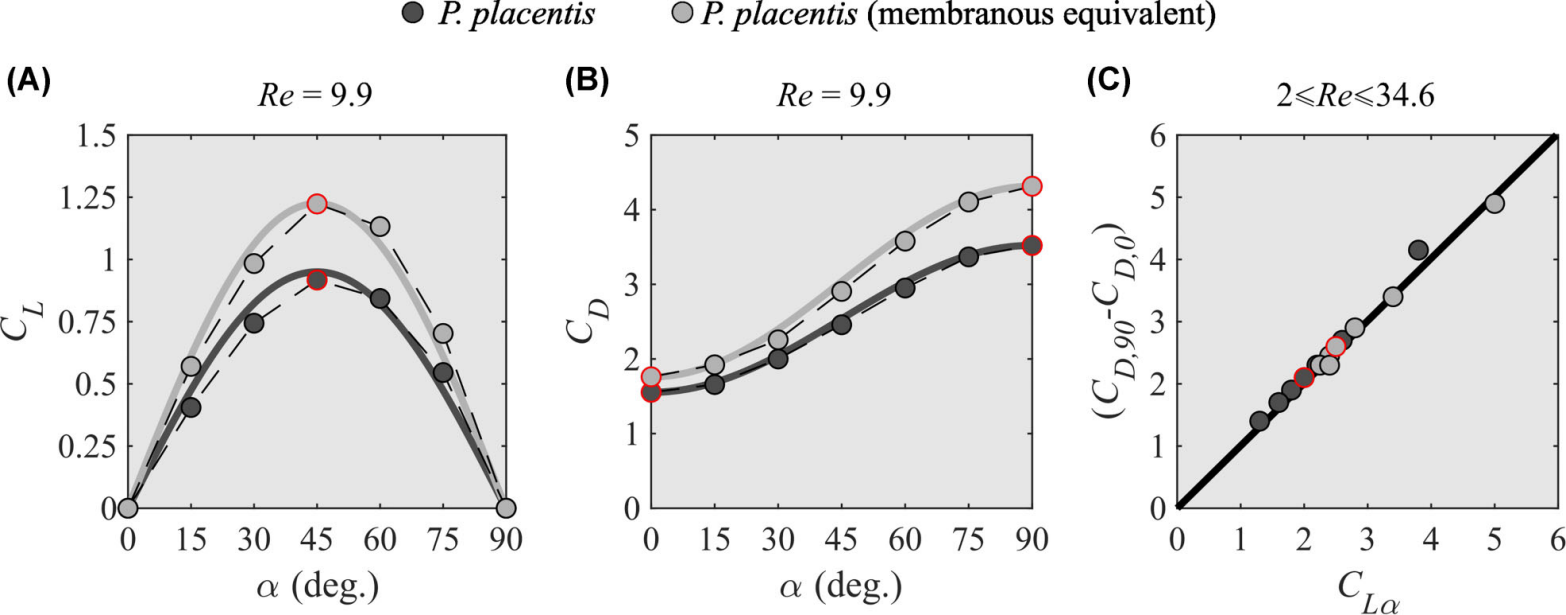
(A) Lift and (B) drag characteristics of the *P. placentis* wing (at $Re=9.9$) across the first quadrant of geometric angle of attack, illustrated by both the experimental data (markers) and the normal force model (solid lines). A similar alignment with the normal force model was revealed for $Re$ values of 2, 4, 5.9, 7.9, 9.9, 19.8, and 34.6. For brevity, we summarize this alignment in (C) via the equality between $C_{L,\alpha }$ and $(C_{D,90} - C_{D,0})$, as according to Equation 10. Panel (C) collates equivalent data [such as those circled in red in (a) and (b)] for $2\le Re \le 34.6$. Red markers in (A) and (B) correspond to the *x*- and *y*-axis values, respectively, of the red markers in (C). The red markers indicate the key data points which can be used to parameterize the normal force model.

**Fig. 7:**
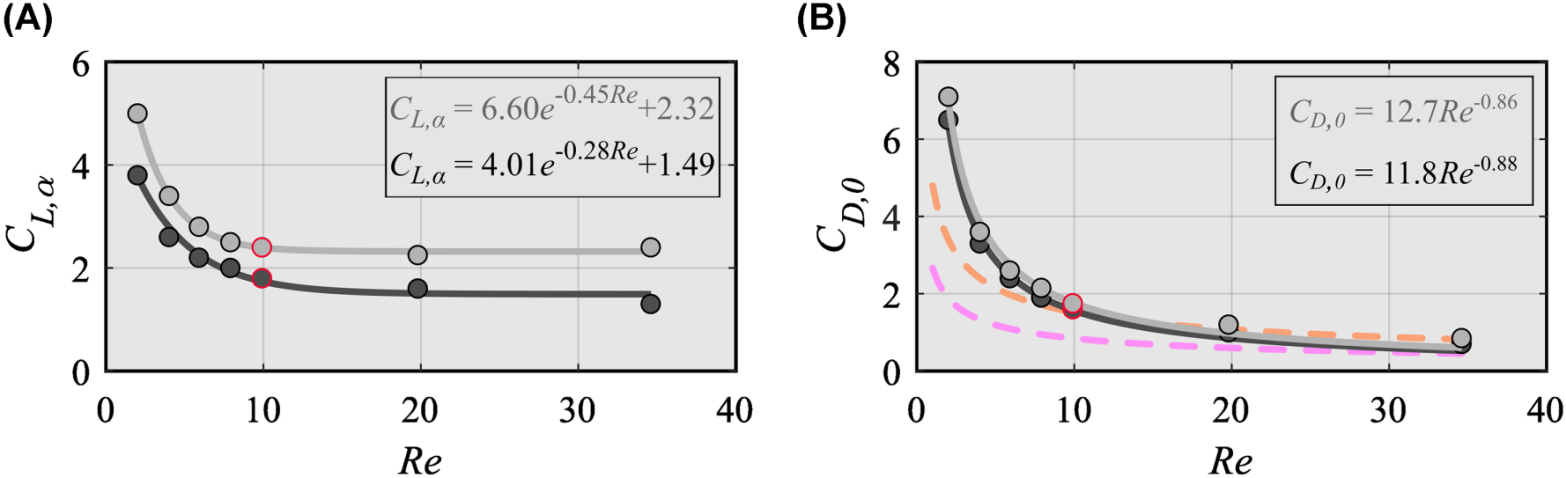
Best-fit relations of (A) $C_{L,\alpha }$ and (b) $C_{D,0}$ against $Re$, based on data sourced from experimental measurements on bristled (dark gray) and membranous (light gray) feathered-winged beetle-like planforms. Best-fit curves are exponential and power functions for $C_{L,\alpha }$ and $C_{D,0}$, respectively. Also included in (B) is the well-known solution for skin friction drag from Blasius (pink) as well as the solution attained by Ellington (orange) in his works on hovering insect aerodynamics (Ellington 1984d), which are given by $2.66/\sqrt{Re_c}$ and $4.8/\sqrt{Re_c}$, respectively.

Across a range of very low $Re$ values (*<*50), Kolomenskiy et al. (2020) experimentally defined drag polars for a dynamically scaled *P. placentis* wing model and its membranous equivalent, while performing continuous revolution (note, the membranous equivalent wing is the same wing with no gaps between the bristles). Figure 6 illustrates some of the lift and drag characteristics extracted from the aforementioned study at $Re=9.9$, in order to demonstrate its alignment with the normal force model. The normal force model is a well-established analytical tool used to model the aerodynamic characteristics of hovering insect wings, assuming that the wing is operating in the absence of classical wing stall. The validity of this assumption will be discussed shortly. It is also assumed that the wing is infinitesimally thin (meaning that there is no chord-wise component to the integrated surface pressure force), the chord-wise tangential force due to skin friction can be accounted for as an additional term in the drag equation, and the magnitude of the normal pressure force is proportional to the projected wing chord perpendicular to the flow direction. The model is parameterized according to the wing’s lift-curve slope ($C_{L,\alpha }$) and zero-lift drag coefficient ($C_{D,0}$) as follows (Broadley and Nabawy 2023):


(9) \begin{eqnarray*}
C_L(\alpha )=C_{L,\alpha }\sin (\alpha )\cos (\alpha ),
\end{eqnarray*}

(10) \begin{eqnarray*}
C_{D}(\alpha ) &=&C_{D,0}+C_{L,\alpha }\sin ^2(\alpha )\\&=&C_{D,0}+(C_{D,90}-C_{D,0})\sin ^2(\alpha ),
\end{eqnarray*}

where $C_L$ and $C_D$ are the lift and drag coefficients during the translational motion, respectively; $\alpha$ is the geometric angle of attack; $C_{L,\alpha }$ is the three-dimensional wing lift curve slope at the vicinity of small angles of attack; $C_{D,0}$ is the zero-lift drag coefficient of the wing (occurring at zero angle of attack when assuming a symmetric wing section, typically a flat plate); and $C_{D,90}$ is the wing drag coefficient at $90^{\circ }$ angle of attack. The normal force model has been widely used for aerodynamic predictions at *Re* values greater than 100 (Broadley and Nabawy 2023) but has not been implemented for cases with *Re* values relevant to that of miniature insects (i.e., $Re< 50$). One of the main assumptions of the normal force model in its current form is that the wing should be operating in the absence of stall (Nabawy and Crowther 2017). In the case of flapping insect wings, this is usually facilitated by the formation of a stably attached LEV. Therefore, if the aerodynamic force coefficients describing a wing align with normal force model, then the above condition can be deemed satisfied. This alignment is shown in Fig. 6A and B for an *Re* of 9.9, where the solid lines illustrate coefficients as according to the normal force model. Kolomenskiy et al. (2020) defined similar data sets for *Re* values ranging from two to 34.6 for which the same alignment with the normal force model can be seen. As it would be tedious to repeat such drag polars for all *Re* values, for brevity, in Fig. 6C, we compare the lift-curve slope values to the difference in the corresponding maximum and minimum drag coefficients of these data sets. The equivalence is evident from the slope of the linear line of best fit (slope = 1.0032), validating the relation $C_{L,\alpha }=\ C_{D,90}-C_{D,0}$, and, hence, providing strong evidence of the validity of Equation 10 of the normal force model. Therefore, it can be concluded that that the normal force model, as described in 9 and 10, can conveniently represent the aerodynamic force coefficients of miniature insect wings operating at *Re* values below 50 against angle of attack, once the values of only two parameters (namely, the lift curve slope, $C_{L,\alpha }$, and zero-lift drag coefficient, $C_{D,0}$) are determined. Also, the particular significance of $C_{D,0}$ in this investigation should be noted. Obviously, $C_{D,0}$ captures the effects of viscous flow effects which, as previously discussed, are very influential in the miniature insect domain. Whereas, for examples of insect flight outside of the miniature domain, the effects of viscosity can be so small that they have been ignored in previous works (Nabawy and Crowther 2015). Of course, as we will see from Fig. 7, $C_{D,0}$ definitely cannot be ignored when considering the aerodynamics of miniature insects.

**Fig. 8:**
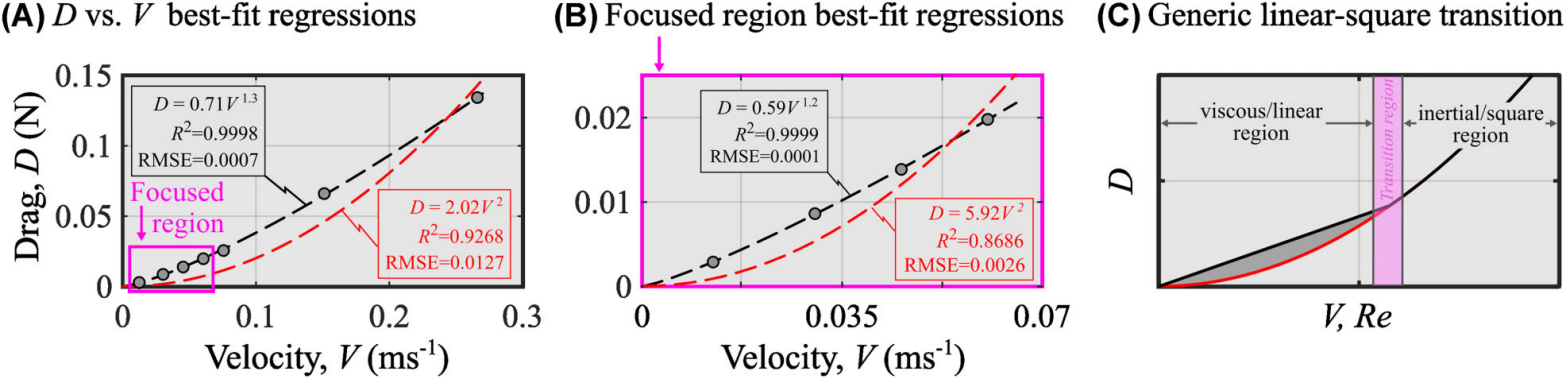
(A) Drag force measurements on a bristled *P. placentis* wing continuously revolving at $\alpha =0^\circ$ at 7 different constant velocities (corresponding to the 7 bristled wing $C_{D,0}$ values in Fig. 7B). Two best-fit regression curves are provided, where the black curve is the *overall* best-fit curve whereas the red curve shows the *quadratic* best-fit curve. Note that the black curve is a near-linear function, highlighting the non-quadratic behavior of the drag force at miniature scales. For completeness, the best-fit relations for the first 4 data points (highlighted by the pink box) are shown in (B) in order to demonstrate that the linearity of the drag behavior increases for lower velocities. In order to explain how the trends defined in (A) and (B) produce those in Fig. 7C, the plot in (C) shows the generic behavior of the drag force as the flow characteristics transition from the Stokes regime (viscous dominated and linear in its function) to the boundary layer regime (inertially dominated and quadratic in its function). The red curve shows the hypothetical drag force if the behavior were to be quadratic in the Stokes regime. It is the disparity between the black linear curve and the red quadratic curve in the viscous region which shows why we see such a significant increase in $C_{D,0}$ (which is calculated in-part via normalization of the drag force by $V^2$) as $Re$ approaches unity in Fig. 7.

Figure 7A and B outlines the $C_{L,\alpha }$ and $C_{D,0}$ values extracted from Kolomenskiy et al. (2020) with respect to $Re$, where the data sets follow exponential and power functions, respectively. Particularly worthy of note, is the behavior of the coefficients for $Re$ values of less than 10. To explain this behavior, it is instructive to remember that the characteristic flows of miniature insect wings are those approaching the Stokes regime, where the fluid dynamic pressure begins to scale with the viscous stresses rather than the inertial stresses. As a result, the scaling of the aerodynamic force components becomes linearly proportional to the translational velocity of the wing (i.e., proportional to *V*), rather than its square (i.e., proportional to $V^2$), since the fluid dynamic forces are no longer dominated by inertial effects. In fact, such behavior can be observed from the *P. placentis* wing data [i.e., that from Kolomenskiy et al. (2020)] by converting the drag coefficients presented in Fig. 7C to the corresponding drag forces in Newtons (see Fig. 8A and B). Note that the drag values have been plotted against *V*; however, the same general trends are revealed when plotted against $Re$, since $Re$ is directly proportional to *V*. The best-fit curves reveal that the variation of the drag force with *V* is near-linear rather than quadratic, and that this linearity increases as *V* is reduced. Furthermore, the generic plot provided in Fig. 8C demonstrates how a linear variation in *D* will result in larger aerodynamic forces than a quadratic variation; ultimately, this disparity leads to the large spikes in the aerodynamic coefficients shown in Fig. 7 as $Re$ approaches unity. What we find is that, when the aerodynamic coefficient is calculated by normalizing the aerodynamic force by $\left(\frac{1}{2}\rho V^2 S\right)$, for a constant air density and wing area we are assuming that the aerodynamic force is proportional to $V^2$ when in fact it is proportional simply to *V*—this leads to huge aerodynamic force coefficients in the low $Re$ region because the force is larger than anticipated by the quadratic assumption. It should be highlighted that the transition between the viscous and the inertial regions is, of course, not immediate and in fact there will be an intermediate transitional region whereby the aerodynamic force scales via neither a linear nor a square relation. We have hence designated a transitional region between the two regimes (see the pink shaded region in Fig. 8C). Notably, the schematic in Fig. 8C is for demonstration purposes only and does not imply the exact nature of the transition. In fact, a potential approximation to capture both the linear and quadratic behaviors simultaneously in one relation is to use a binomial scaling (i.e., $D \propto C_1 V + C_2 V^2$) that ensures the correct asymptotic behavior. It must also be addressed that we have directed the above discussion to drag and the drag coefficient and that it is not particularly obvious why $C_{L\alpha }$ seems to scale according to an exponential function rather than the power function.

Notably, the lift curve slope data in Fig. 7A is represented with exponential relations. While this mathematical representation provides an excellent fit for the data, there are also other mathematical formulae that can equally provide a satisfactory representation, particularly the binomial form: $C_{L,\alpha } = A_1 / Re + A_2$. Similarly, the zero-lift drag coefficient data in Fig. 7B can also be satisfyingly represented via the binomial form: $C_{D,0} = B_1 / Re + B_2 / \sqrt{Re}$.

In the context of classic insect wing aerodynamics, it could be reasoned that the enhanced effects of viscous flows may lead to diminished lift-characteristics for lower $Re$ operations and that the trends shown in Fig. 7A are somewhat counter-intuitive. We say this because vortical structures play a significant role in the high performance of flapping winged insects, but they may become diminished as viscous flow effects dissipate their energy with greater effect at lower $Re$. It is well-known that the LEV significantly contributes to the remarkable performance of insects, hence, we might be tempted to suggest that a wing’s lift coefficients will diminish if the LEV is dissipated via heightened shear reaction to circular flows. Conversely, since we are considering flows which are approaching the Stokes regime, the high influence of flow viscosity will likely help to prevent flow separation and may render the role of the LEV redundant.

The above discussions are made based upon the limited amounts of existing literature available at present and require further empirical investigation to uncover their true nature. With that being said, there does exist some related experimental findings which show that the vortical structures on the leading- and trailing-edges of a flapping bristled wing at $Re= 3.4$ are rapidly dissipated during stroke reversal at $Re$ values below 10 (O’Callaghan and Lehmann 2023). O’Callaghan and Lehmann (2023) also found that there was no evidence of the Wagner effect throughout the flapping half-stroke and that the starting vortex hadn’t completely shed from the trailing edge by the end of a flapping half stroke. Moreover, Santhanakrishnan et al. (2018) showed that for a steadily revolving elliptical wing, after a 90$^\circ$ of the revolution both the leading- and trailing-edge vorticity remained attached to the wing within the range $4 \le Re \le 32$, whereas, for $Re = 2$ and $Re = 1$ there wasn’t clear evidence of a leading- or a training-edge vortex having been generated. The reader is referred to the review by Sidebottom et al. (2025b) for further discussions on miniature-scale flow effects and aerodynamic mechanisms. Overall, however, it is important to note that the performance of miniature insect wings might not be as significantly attributed to flow vorticity and the LEV as they are for their larger counter parts. In fact, below $Re \approx 10$, the nature in which aerodynamic force is generated on miniature insect wings might be different from the classical explanation relating to the generation of a stably attached LEV. With that being said, miniature insects are typically expected to operate at $Re > 10$ and so the conventional explanation of insect aerodynamics may very well still be appropriate.

As mentioned, Fig. 7B outlines $C_{D,0}$ as a function of $Re$ for the *P. placentis* wing. Also included in the figure is the well-known Blasius solution for $C_{D,0}$, describing the incompressible two-dimensional flow over a flat plate ($2.66/\sqrt{Re}$), as well as the solution determined by Ellington (1984d) describing *Drosophila* wings ($4.8/\sqrt{Re}$). The agreement between those additional solutions and the solutions related to the *P. placentis* wing is notable, particularly for $Re$ values greater than 20. However, for $Re$ values less than 20 and 10, it appears that the skin friction predictions made by Blasius’ and Ellington’s solutions, respectively, begin to under-estimate those found for the *P. placentis* wing. It should be noted that Ellington defined the above relationship using data describing a *Drosophila* wing performing linear translation, whereas the results extracted for the *P. placentis* wing were generated using a continuously revolving wing. Additionally, Ellington’s solution was defined for a $Re$ range outside of that of the miniature domain, hence his curve illustrated in Fig. 7B can be considered an extrapolation.

Now that the aerodynamic coefficient relations have been outlined, it is instructive to highlight how they will constrain the parameter space range for the analysis that we carry out in the rest of the study. Here, we define *Re* as


**Fig. 9:**
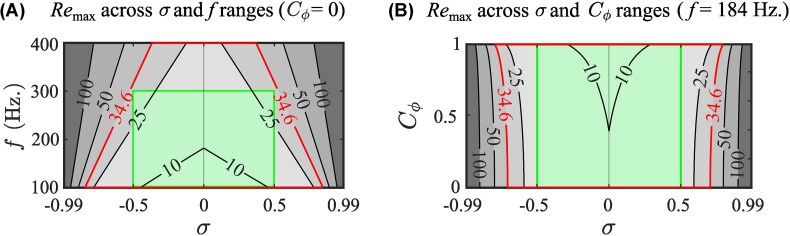
Maximum $Re$, $Re_{\mathrm{max}}$, throughout entire stroke as according to variations in $\sigma$ and either (A) *f* or (B) $C_{\phi }$. In (A) $C_\phi$ is given a representative value of 0, since that is the control parameter setting which causes the largest value of $Re_{\mathrm{max}}$ for a given $\sigma$ [as can be seen in (B)], and hence provides us with the real edge-case value of $Re_{\mathrm{max}}$ for a given *f*. While, in (B), *f* has been given a representative value of 184 Hz, which is the flapping frequency extracted for the miniature featherwinged beetle (see Table 1). The red contour is $Re=34.6$ which is the maximum $Re$ for which the extracted aerodynamic coefficients $C_{L,\alpha }$ and $C_{D,0}$ are defined (see Fig. 7). Hence, the valid parameter space ranges across which the variables $\sigma$, *f*, and $C_{\phi }$ can be defined, exist inside of the red bounded region on each plot. In the present study, the chosen maximum stroke asymmetry range was $-0.5\le \sigma \le 0.5$ which allows for safe assessment of *f* and $C_{\phi }$ values across the ranges 100 Hz $\le f \le$ 300 Hz and 0 $\le C_{\phi } \le$ 1, respectively (outlined by the green box).

(11) \begin{eqnarray*}
Re = \frac{\rho V \bar{c}}{\mu },
\end{eqnarray*}

where $\rho$ is the air density (assumed to be that under standard sea level conditions, $\rho$ = 1.225 kg/m$^3$), *V* is the translational wing velocity (as defined by Equation 3), and *μ* is the dynamic air viscosity (defined as *μ* = 1.81 $\times 10^{-5}$ Pa s, characteristic of atmospheric pressure conditions at 20$^\circ$C). As implied by Equation 11, for a given wing geometry operating in air, the value of $Re$ is solely dependant on the translational velocity of the wing. In this investigation, for a fixed stroke amplitude, the translational velocity of the wing is controlled by three parameters only: $\sigma$, *f*, and $C_{\phi }$, all of which are used to define the parameter space range in our investigation. Therefore, it is necessary to determine which values of $\sigma$, *f*, and $C_{\phi }$ lead to $Re < 34.6$, since this is the greatest $Re$ value used to define the aerodynamic relations provided in Fig. 7. We do not wish to exceed $Re = 34.6$ since this would involve extrapolating beyond the boundaries of the known aerodynamic characteristics. Hence, in accordance with the results provided in Fig. 9 for $Re_{\mathrm{max}}$, in this investigation, our chosen maximum stroke asymmetry range is $-0.5 \le \sigma \le 0.5$, which allows us to safely assess *f* and $C_{\phi }$ values across the ranges $100\, \mathrm{Hz.} \le f \le 300\, \mathrm{Hz.}$ and $0 \le C_{\phi } \le 1$, respectively. These boundaries are highlighted via the green boxes in Fig. 9. It is true that our selected limits for $\sigma$ are not the only viable options for our study and that there are other ranges that are both larger and smaller than $-0.5 \le \sigma \le 0.5$ which could be considered. However, we chose this particular range because it embodies a neat and comprehensive absolute $\sigma$ variation of 1.

As a final point, as per the above discussion is it important to remember that throughout a wing stroke the characteristic $Re$ will vary between a value of 0 (when the wing is stationary during stroke reversal) and, depending on the values of $\sigma$, *f*, and $C_\phi$, a certain value of $Re_{\mathrm{max}}$. Considering the values of $Re_{\mathrm{max}}$ presented in Fig. 9, it is therefore safe to say that $C_{L,\alpha }$ and $C_{D,0}$ will vary significantly throughout the wing stroke as the flow characteristics vary across the range $0 \le Re \le Re_{\mathrm{max}}$ (as according to the trends defined in Fig. 7). For instance, suppose $Re_{\mathrm{max}}=34.6$, across the range $0 \le Re \le 34.6$ the characteristic $C_{L,\alpha }$ for a bristled wing varies across $5.5 \le C_{L,\alpha } \le 1.49$ (see Fig. 7). Such a significant change in aerodynamic coefficient is unprecedented across a single wing stroke, especially when considering the flight of non-miniature insects (Lentink and Dickinson 2009). As such, in this investigation the normal force model has been defined not only as a function of $\alpha$ but also as a function of $Re$. It is through the above discussion that the uniqueness of the miniature flight category becomes most apparent. The associated aerodynamics can vary significantly through only slight alterations in the wing velocity, particularly those corresponding to $Re \le 10$, in a way that is not true for larger flying creatures or aircraft.

### Quasi-steady aerodynamic framework

Expressions for the aerodynamic lift and drag coefficients, ($C_L$ and $C_D$, respectively), were defined using Equations 9 and 10, with the lift curve slope and zero-lift drag coefficient values being evaluated based on the best-fit relations displayed in Fig. 7. Hence, deploying Equations 9, 10, 3, and 8 within the classical definitions of lift, drag, and aerodynamic power, the time-averaged values across a full stroke can be written as


(12) \begin{eqnarray*}
\bar{L}=K_L \int _{0}^{T} C_{L,\alpha }(t) \Bigl ( V{\mid _{\hat{r}_2}}(t)\Bigl )^2\sin {\bigl (\alpha (t)\bigl )}\cos {\bigl (\alpha (t)\bigl )} \, dt,\\
\end{eqnarray*}

(13) \begin{eqnarray*}
\bar{D}&=&K_D \Biggr [ \Biggl (\int _{0}^{T} C_{D,0}(t) \Bigl (V{\mid _{ \hat{r}_2}}(t)\Bigl )^2 \, dt \Biggl )\\&&+ \Biggl (\int _{0}^{T} C_{L,\alpha }(t) \Bigl (V{\mid _{ \hat{r}_2}}(t)\Bigl )^2 \sin ^2\bigl (\alpha (t)\bigl ) \, dt \Biggl )\Biggr ],
\end{eqnarray*}

(14) \begin{eqnarray*}
\bar{P}&=&K_P \Biggr [ \Biggl (\int _{0}^{T} C_{D,0}(t) \Bigl (V{\mid _{ \hat{r}_3}}(t)\Bigl )^3 \, dt \Biggl )\\&&+ \Biggl ( \int _{0}^{T} C_{L,\alpha }(t) \Bigl (V{\mid _{ \hat{r}_3}}(t)\Bigl )^3 \sin ^2\bigl (\alpha (t)\bigl ) \, dt \Biggl )\Biggr ],
\end{eqnarray*}

where,


(15) \begin{eqnarray*}
K_L=K_D=\frac{1}{2}{\rho }f(2R\bar{c}),
\end{eqnarray*}

(16) \begin{eqnarray*}
K_P=\frac{1}{2}{\rho }f(2R\bar{c}),
\end{eqnarray*}

where *T* is the time period of a full stroke, which is the reciprocal of the flapping frequency, *f*, and $\rho$ is the air density. In the above expressions, the aerodynamic coefficients are presented a function of time because they are defined as a function of $Re$ which varies throughout the flapping cycle. The first term of the expression for aerodynamic drag (Equation 13) accounts for the skin friction contribution (typically neglected for large insects, but dominant for miniature insect flight), whereas the second term accounts for pressure and induced drag contributions. The notations $V{\mid _{ \hat{r}_2}}$ and $V{\mid _{ \hat{r}_3}}$ in Equations 12–14 refer to the wing’s translational velocity at time *t* when calculated with resect to either $\hat{r}_2$ or $\hat{r}_3$ depending on whether aerodynamic force or aerodynamic power is being calculated, respectively. It should also be highlighted that the total wing area (i.e., $2R\bar{c}$) describes the theoretical fully membranous wing area (i.e., equivalent to a bristled wing with no spaces between the bristles) rather than the surface area of the bristled wing. It is already well established that the bristled wings of miniature insects can generate between $70\, {\rm and}\, 80\%$ of the aerodynamic force generated by their membranous equivalents (Barta and Weihs 2006; Davidi and Weihs 2012; Kasoju et al. 2018; Kolomenskiy et al. 2020; Jiang et al. 2022; Sidebottom et al. 2025b) owing to the shear layers that form either side of the bristles and cause the gaps between them to become partially blocked. As such, in this study, we consider the bristled wings to *behave* as though they are fully membranous and therefore we refer to the membranous wing area rather than the bristled wing area.

## Model verification

The outlined quasi-steady framework is validated against numerical data from Cheng and Sun (2018) in which a miniature wasp wing performing a pure lift-based stroke was assessed for its lift and drag performance numerically—see Table 2 for the extracted input parameters. The extracted numerical results are for a flapping wing only, and corresponding translational coefficients describing the wasp wing at miniature scale $Re$ (i.e., such as those presented in Fig. 7) are not available. Hence, to mitigate the absence of appropriate translation aerodynamic data, we performed validation via three approaches which simply involved using three different methods of parameterizing the normal force model.

**Table 2:** Geometric and kinematic parameters used to parameterize the quasi-steady model for the validation study

Parameter	*R* (mm)	$\bar{c}$ (mm)	$\hat{r}_{2}$ (mm)	*f* (Hz)	$\phi _{\mathrm{amp}} (^\circ )$	$\theta _{\mathrm{amp}} (^\circ )$
Value	0.61	0.38*R*	0.64*R*	361	72	45

*Note*: The parameters describe a miniature wasp wing that was assessed while performing pure lift-based kinematics in a previous numerical study (Cheng and Sun 2018).

First, the aerodynamic relations defined in Fig. 7 were used to parametrize the normal force model, causing errors in the time-averaged lift and drag forces of 3.70 and −31.3%, respectively. The much larger error for the drag force estimates can potentially be explained by the phenomena identified by Sidebottom et al. (2025b) in which the translational aerodynamic data in Fig. 7 was compared to that from a CFD assessment of a membranous elliptical wing planform (Santhanakrishnan et al. 2018) across a similar $Re$ range. For the reader’s convenience, the aerodynamic relations describing the referenced elliptical wing study are as follows:


(17) \begin{eqnarray*}
C_{L,\alpha }=5.57e^{-0.36Re}+2.67,
\end{eqnarray*}

(18) \begin{eqnarray*}
C_{D,0}=20.8Re^{-0.73}.
\end{eqnarray*}

Note that Equations 17 and 18 are curve fitting expressions defined by the authors of the present investigation and are valid across the range $4 \le Re \le 64$. As can be expected from a surface level inspection of the above relationships, the comparison between them and the experimental relations given in Fig. 7 revealed a much larger disparity in the skin friction values (with the CFD estimations being much larger than the experimental) than the lift-curve slope values (which were in reasonable agreement with one another).

**Fig. 10:**
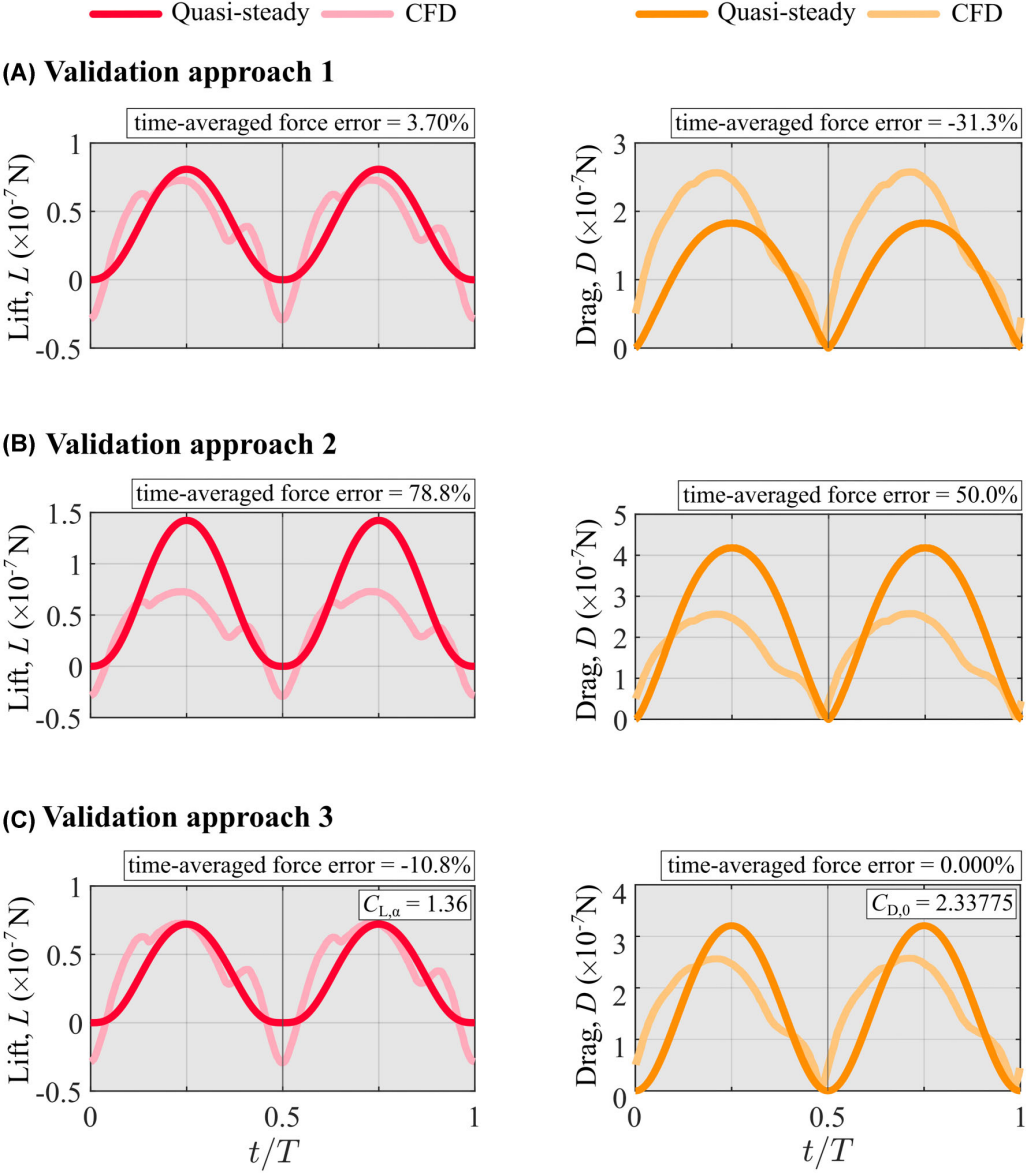
Quasi-steady lift and drag force estimates of a flapping miniature wasp wing performing pure lift-based kinematics, superimposed with numerical data describing the same wing performing the same kinematics similar to that shown in Fig. 5 for $\theta _{\mathrm{amp}}=45^\circ$. Dark red and orange data curves represent the calculated quasi-steady lift and drag forces, respectively, while lighter shaded red or orange curves represent the lift and drag forces extracted from the referred numerical study, respectively. The quasi-steady results in (A) were obtained using the experimental translational force coefficient relations outlined in Fig. 7 to parameterize the normal force model. For the quasi-steady results presented in (B), the normal force model was parameterized using the numerical expressions extracted from Santhanakrishnan et al. (2018), describing the translational lift-curve slope and skin friction components for a membranous elliptical wing revolving at miniature scale $Re$ values across the range $4 \le Re \le 64$. Finally, in (C), the model was parametrized using the mid-half-stroke lift-coefficient from the validation data (i.e., that at $t/T=0.25$) to approximate the lift-curve slope for the normal force model via the approximation $C_{L,\alpha }=2C_{L,max}$. Then, the appropriate $C_{D,0}$ value was extracted in order to reduce the overall percentage error to 0% (i.e., $C_{D,0}=2.33775$).

Hence, moving on to the second validation approach (see Fig. 10B), considering the disparity described above, it seemed appropriate to also try parameterizing the normal force model as according to Equations 17 and 18, since the validation data itself is also numerical (see Fig. 10bB). However, the second approach resulted in much larger errors in both lift and drag (78.8 and 50.0%, respectively). For the final validation approach, $C_{L,\alpha }$ was defined as 1.36 which is double that of the $C_L$ value in the validation data at the half mid-stroke when the wing is at $\alpha =45^\circ$. This value allows the quasi-steady lift model to coincide with the validation data at the mid half-stroke. $C_{D,0}$ was then defined as 2.33775 such that the time-averaged error of the quasi-steady drag model was reduced to 0%.

At this point it must be said that, while the quasi-steady model somewhat struggles to accurately predict the drag behavior of the miniature wasp wing, this struggle is a symptom of the field as a whole rather than just one of the quasi-steady assumption—with there being clear disagreements across all investigation types. Whether that disagreement is caused by differences in wing planforms, methods of investigation (i.e., experimental or numerical), or the considered aerodynamic mechanisms, is yet to be revealed owing to the infancy of the miniature insect aerodynamic field. However, as discussed in Section “Measures of aerodynamic performance”, it should be emphasized that, while the provided validation is a complementary feature of this article, the purpose of the present investigation isn’t to provide detailed evaluations of the aerodynamic characteristics of miniature insect wings. Rather, the intention is to generate quick estimates of such characteristics in order to reveal the overall aerodynamic trends which occur as a result of introducing stroke asymmetry and altering the flapping and pitching waveforms. With all of the above considered, the validation provided here is considered sufficient to justify the use of the quasi-steady framework for its intended use in this study.

## Measures of aerodynamic performance

The ability for a flying body to remain airborne is largely dependent on the amount of aerodynamic force that it can generate in the direction opposing that of the gravitational force. In the present study, we will refer to said aerodynamic force as the “vertical aerodynamic force”, denoted by ${F}_{V}$ (or $\bar{F}_V$, when time-averaged across a full wing stroke). Naturally, for the pure lift- and drag-based profiles defined in Section “Motion representation”, $F_V$ will be composed of contributions from *only* either the wing’s lift or drag force, respectively, depending on which style is being considered. As such, to distinguish between the lift- and drag-based styles, when referring to the vertical aerodynamic force, we will use $F_{V,L}$ and $F_{V,D}$, respectively. Additionally, for wing strokes which generate greater values of $\bar{F}_{V}$, we say that the stroke is more “effective”. Hence, the metric of “effectiveness” is used throughout this paper to describe wing strokes that generate either greater or smaller values of $\bar{F}_{V}$ than other strokes.

As touched upon in Section “Introduction”, for a meaningful assessment of a flying body’s aerodynamics, it is also necessary to measure its aerodynamic efficiency in some fashion. In the present study, we use three metrics of aerodynamic efficiency: the reciprocal of the aerodynamic power required to generate a specific aerodynamic load, $1/P_{hov}$; the glide ratio, $GR$; and the power factor, $PF$. It should be noted that $P_{hov}$ is inversely proportional to aerodynamic efficiency, since it is more optimal to minimize the aerodynamic power expenditure required to generate a specific aerodynamic load. Hence, it is convenient to take the reciprocal of $P_{hov}$, rather than simply the raw value alone, since $1/P_{hov}$ will increase or decrease with increasing or decreasing aerodynamic efficiency, respectively. The aerodynamic power required for hovering flight is calculated via substitution of insect weight (see Table 1) into Equation 12 or 13 (for lift- or drag-based flight, respectively) via $\bar{L}=W$ or $\bar{D}=W$ and rearranging to solve for *f*. The calculated value of *f* corresponds to the flapping frequency required for hovering flight, $f_{hov}$, which, when substituted in to Equation 14, provides $P_{hov}$. Usually, $f_{hov}$ will be determined analytically, however, due to the new nature of the current quasi-steady framework ensuring that the normal force model is a function of $Re$ as well as $\alpha$ (see Section “Aerodynamic coefficients”), $f_{hov}$ was determined numerically using the Newton–Raphson convergence method. With regards to $GR$ and $PF$, in the context of flapping wing flight, $GR$ is the non-dimensional aerodynamic ratio of the weight that a wing can support for a unit *drag*, whereas $PF$ is the non-dimensional aerodynamic ratio of the weight that a wing can support for a unit *power* (Kruyt et al. 2015)—calculated via $GR=C_L/C_D$ and $PF = C_L^{3/2}/C_D$, respectively.

## Results

### Overview

In Section “Aerodynamic coefficients”, we highlighted the importance of considering temporal values of the aerodynamic coefficients $C_{L,\alpha }$ and $C_{D,0}$ when modeling miniature insect aerodynamics via the quasi-steady approach. Hence, in this section, we begin by presenting the results as corresponding to aerodynamic coefficients which vary not only as according to the geometric angle of attack, but also to the local chord-based $Re$ at each stage of the wing stroke. The temporal variation of the aerodynamic coefficients is captured in Equations 12–14 where the $C_{L,\alpha }$ and $C_{D,0}$ terms appear inside of the integral rather than as constants. For completeness, we also wish to demonstrate the implications of modeling miniature insect aerodynamics in this manner by providing a comparison to the traditional approach, whereby the aerodynamic coefficients are assumed to be dependent on only the wing’s angle of attack (i.e., where $C_{L,\alpha }$ and $C_{D,0}$ are fixed values). As such, the results provided in Sections “Aerodynamic performance for symmetric profiles” and “Considering stroke asymmetry” are concerned with $Re$-dependent values of $C_{L,\alpha }$ and $C_{D,0}$, whereas Section “Assessing the implications of using *Re*-dependant aerodynamic coefficients” provides a comparison of those results to results where $C_{L,\alpha }$ and $C_{D,0}$ are taken as fixed values corresponding to $Re=10$ (i.e., the typical $Re$ value used to characterize the miniature flight domain).

### Aerodynamic performance for symmetric profiles

**Fig. 11:**
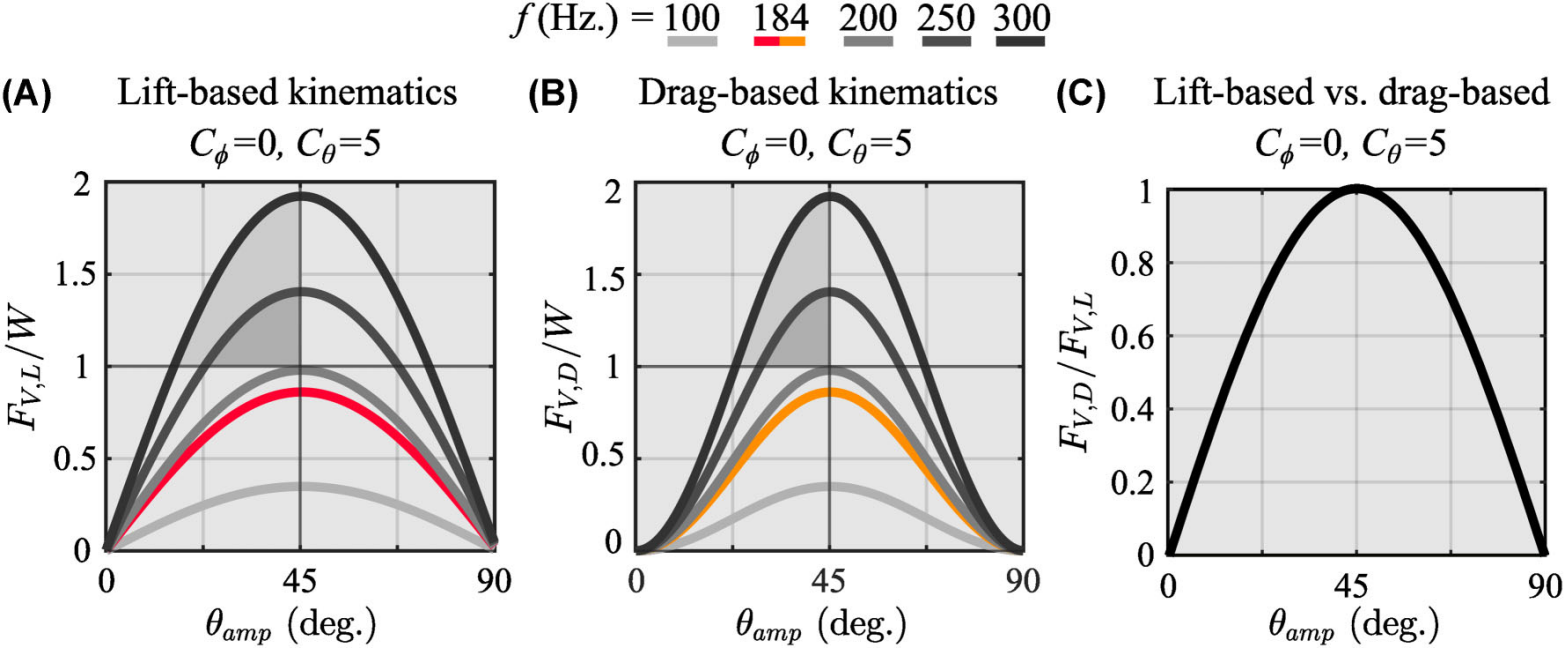
The time-averaged vertical aerodynamic force generated across a single stroke for (A) lift- and (B) drag-based kinematics. The results are associated with $C_\phi$ and $C_\theta$ values of 0 and 5, respectively (which is the well-known condition for generating maximum aerodynamic lift) and *f* values of 100, 184, 200, 250, and 300 Hz (where 184 Hz is the value extracted for the *P. placentis*, see Table 1). Dark gray shaded regions (i.e., darker than the plot background shade) represent ranges within which an insect can generate sufficient vertical force for hovering flight (i.e., higher than its weight). The curve in (C) illustrates the time-averaged aerodynamic force generated across the drag-based stroke as a fraction of that across the lift-based stroke (i.e., $\bar{F}_{V,D}/\bar{F}_{V,L}$), for specific $\theta _{amp}$ values. The shape of the curve in (C) is owing to the fact that drag-based kinematics are governed by a $\sin ^2{(\alpha )}$ relationship (see Equation 10), whereas lift-based kinematics are governed by a $\sin {(\alpha )}\cos {(\alpha )}$ relationship (see Equation 9).

Assessment of the quasi-steady model for symmetric half-strokes was carried out for $\theta _{amp}$ values across the first quadrant; *f* values ranging 100–300 Hz; and the control parameters $C_\phi$ and $C_\theta$ values ranging 0–1 and 0–5, respectively. Figure 11A and B illustrate the effectiveness of lift- and drag-based translational profiles, respectively, normalized with respect to insect weight (as defined in Table 1). The outputs correspond to strokes that are symmetric (i.e., $\sigma = 0$), with sinusoidal and rectangular translational and pitching waveforms, respectively (i.e., when $C_{\phi }$ and $C_{\theta }$ are 0 and 5, respectively), which are the well-established conditions for maximum effectiveness (Nabawy and Crowther 2015). For both lift- and drag-based kinematics when $\theta _{amp}=45^\circ$, hovering flight becomes feasible for operations where *f* is greater than approximately 200 Hz, where smaller $\theta _{amp}$ values become sufficient for weight balance in hovering flight as *f* becomes larger.

Figure 11C illustrates a comparison between lift- and drag-based kinematics via the ratio between the effectiveness of drag-based kinematics and that of lift-based kinematics (i.e., $\bar{F}_{V,D}/\bar{F}_{V,L}$) for $\theta _{amp}$ values across the first quadrant. A significant result is that which occurs when $\theta _{amp}$ is $45^\circ$, when the effectiveness of the lift- and drag-based styles coincide. Then, most surprisingly, Fig. 11C shows that, generally speaking, lift-based kinematics are the more effective method of flight at miniature scales when considering strokes that are symmetric about the half-period.

**Fig. 12:**
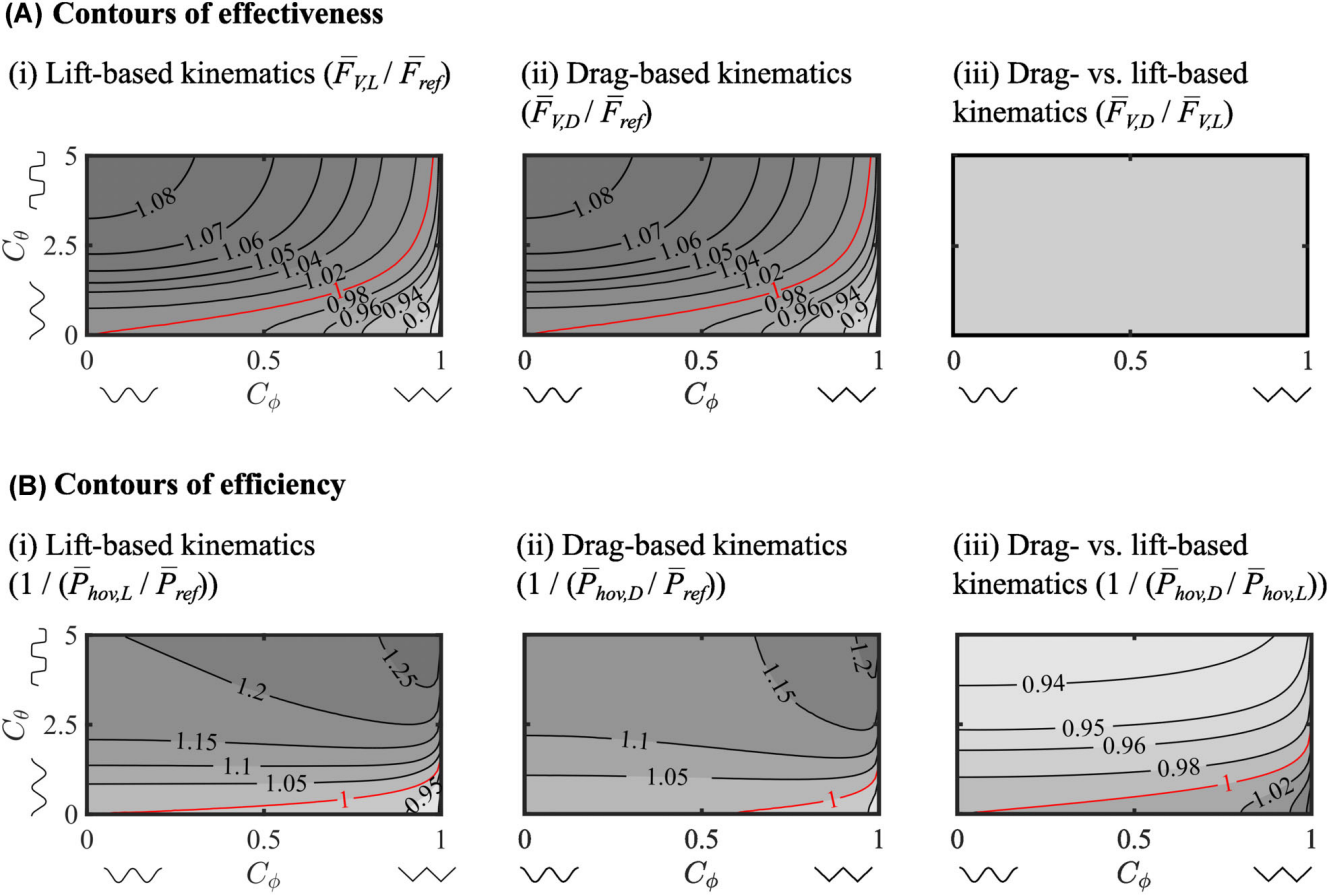
Results for symmetric half-stroke periods. Variations in (A) aerodynamic effectiveness (where $f=184$Hz) and (B) aerodynamic efficiency (i.e., $1/P_{hov}$). In both (A) and (B), plots (i) and (ii) are associated with lift-based kinematics and drag-based kinematics, respectively, while (iii) provides a comparison between the two styles, via either the ratio between their aerodynamic effectiveness or their aerodynamic efficiency, for a particular $C_\phi$ and $C_\theta$ combination. In each plot, values have been normalized with respect to either $\bar{F}_{\mathrm{ref}}$ and $\bar{P}_{\mathrm{ref}}$ which correspond to the vertical force and power for hovering flight, respectively, for sinusoidal translational and pitching variations (i.e., when $C_\phi = C_\theta = 0$). Note that darker shades of gray indicate more desirable outputs. This demonstration is based on the numerical integration of Equations 12–16 with a $\theta _{amp}$ value of $45^\circ$ (i.e., the condition where lift- and drag-based kinematics coincide—see Fig. 11). In (A)(iii), the lack of contours represents an even spread across the parameter space range owing to the fact that plots (A)(i) and (ii) are identical.

Figure 12A and B shows the variations in effectiveness and aerodynamic efficiency, respectively, for lift- and drag-based kinematics for $f=184$ Hz, $\sigma = 0$, and $\theta _{\mathrm{amp}}=45^\circ$. The variations are defined as according to changes in $C_\phi$ and $C_\theta$ across the ranges 0–1 and 0–5, respectively, and the results are normalized with respect to the values for sinusoidal translational and pitching angle variations (i.e, $C_{\phi }=C_{\theta }=0$). The contours of effectiveness are identical for symmetrical lift- and drag-based kinematics and the trend exactly matches the well-established understanding that effectiveness is maximized for sinusoidal and rectangular translational and pitching variations, respectively (Nabawy and Crowther 2015). As such, as far as symmetric half-strokes are concerned, lift- and drag-based kinematics are identical with regards to effectiveness. However, there is a minor disparity between the two flapping profiles when considering aerodynamic efficiency (see Fig. 12B). It is evident from Fig. 12B(iii) that while lift- and drag-based kinematics are identical for sinusoidal translational and pitching variations, lift-based kinematics become the more efficient style as the pitching waveform becomes more rectangular (area above the red reference line). On the other hand, as the translational waveform becomes more triangular, drag-based kinematics begin to become more efficient (area below the red reference line); however, this occurrence is generally for lower $C_\theta$ values. With that being said, the general trends illustrated in Fig. 12B(i) and (ii) are the same for both lift- and drag-based kinematics, and, again, these do not deviate away from the status quo for flapping flight (Nabawy and Crowther 2015). The above findings are intriguing, since they allude to the idea that the lift-based approach is the more favorable one at miniature scales in some scenarios, rather than one that is purely drag-based. However, as already discussed, the pure-drag-based stroke is not completely representative of swimming-like miniature-scale flight, yet the findings still encourage us to question why we are yet to observe lift-based flight in the miniature domain. In the next subsection, we introduce stroke asymmetry in order investigate this point further, with a particular focus on the effects of skin friction drag.

**Fig. 13:**
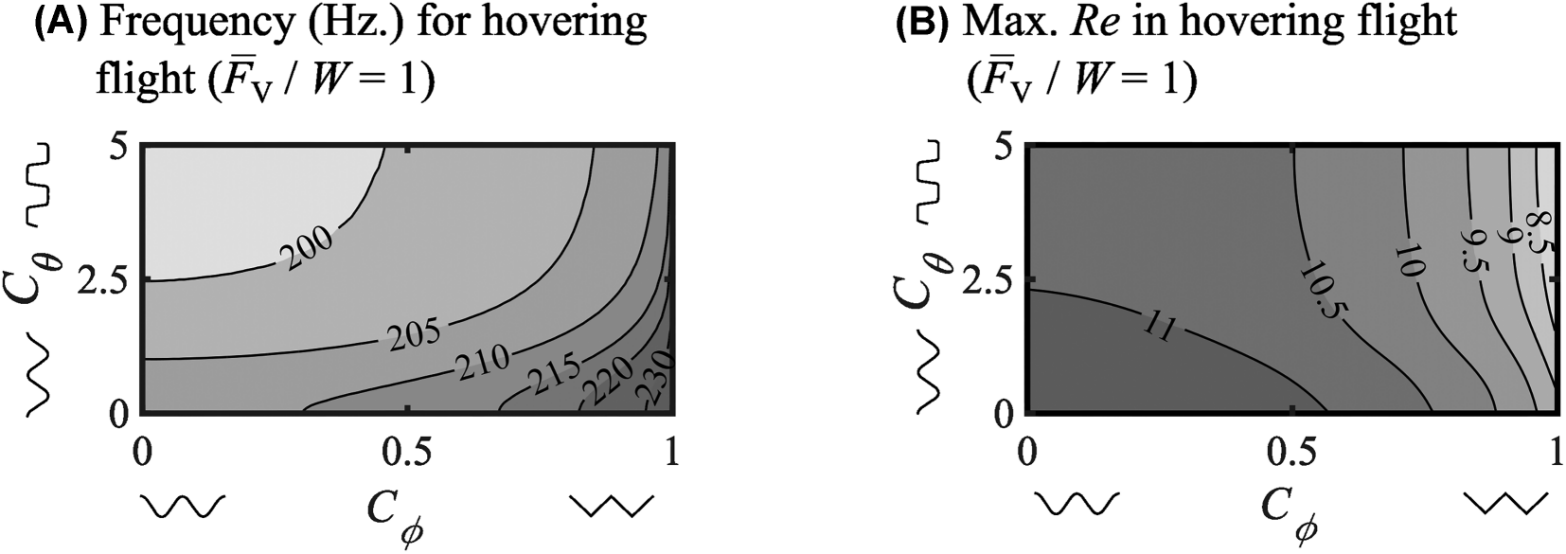
Variations in (A) flapping frequency for hovering flight, and (B) $Re_{\mathrm{max}}$ in hovering flight. This demonstration is based on the numerical integration of Equations 12–16 with a $\theta _{amp}$ value of $45^\circ$ (i.e, the condition where lift- and drag-based kinematics coincide—see Fig. 11).

For completeness, Fig. 13A shows the flapping frequencies required to achieve hovering flight for various translational and pitching waveforms. Notably, the flapping frequency contours are identical for pure lift- and drag-based flight. Fig. 13B shows the corresponding $Re_{\mathrm{max}}$ value for each translational and pitching waveform combination in hovering flight, illustrating how the $Re$ is greatest for sinusoidal waveforms and reduces by a value of approximately 3 as the translational and pitching waveforms become triangular and rectangular, respectively.

**Fig. 14:**
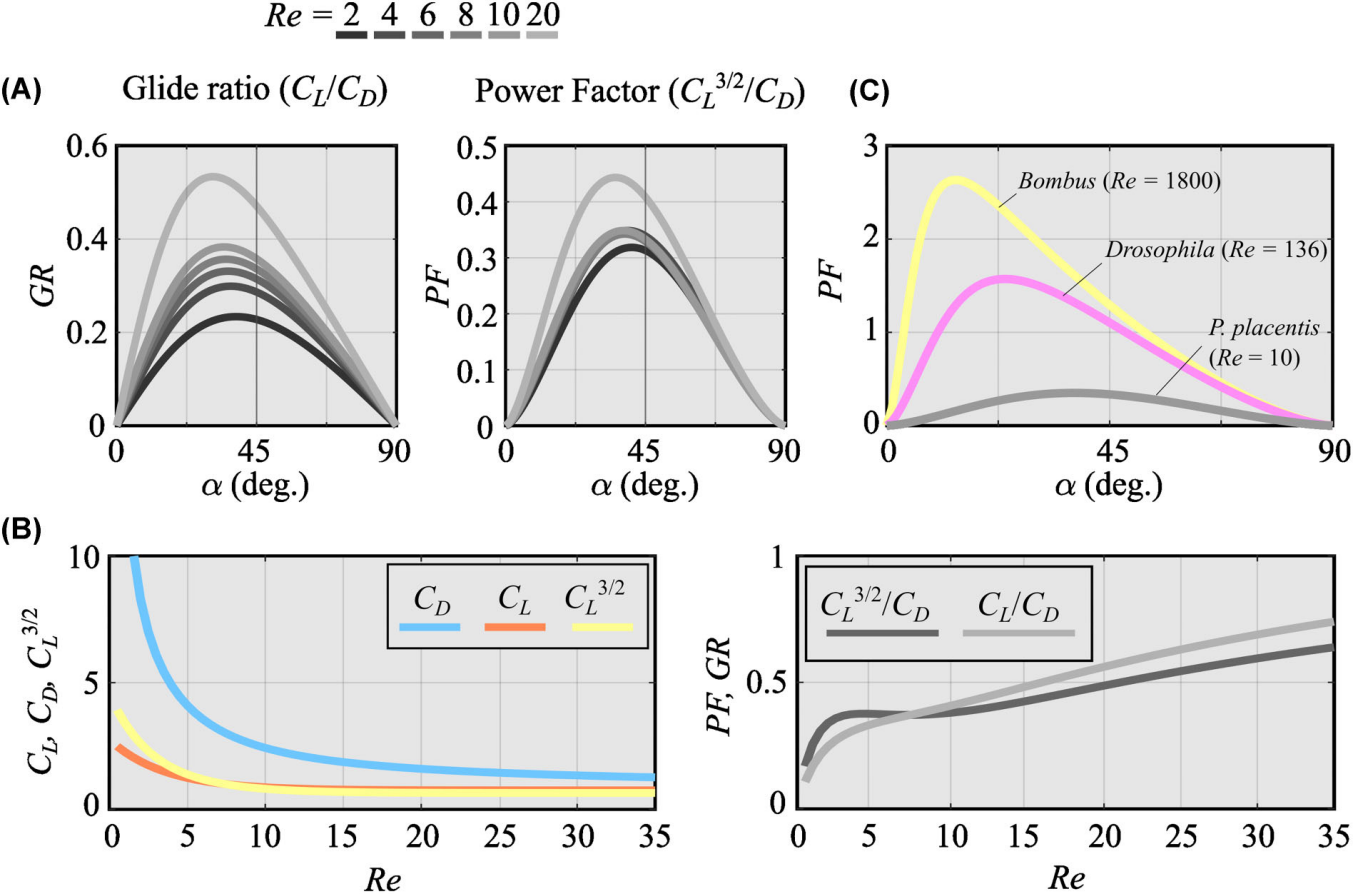
(A) Glide ratio and power factor vs. angle of attack for the *P. placentis* wing at various *Re*. $GR$ and $PF$ were calculated based on the extracted lift-curve slope and zero-lift drag coefficient definitions (see Fig. 7) when deployed into Equations 9 and 10. (B) Variations in $C_L$, $C_D$, $C_L^{3/2}$, $PF$, and $GR$ as according to $Re$ when $\alpha =45^\circ$. The purpose of this figure is to show why a number of the $PF$ curves in (A) are clustered together around the region of $4< Re< 10$, while the $GR$ creates a much more even distribution with $Re$. Ultimately, this clustering effect is simply the result of the curve fittings defined in Fig. 7. (C) A $PF$ comparison of the *P. placentis* to other flapping wing organisms outside of the miniature domain, including the *Bombus* (Wu and Sun 2004), and the *Drosphila* (Dickinson et al. 1999). In a similar fashion, the $PF$ curves for the larger organisms were defined by extracting the lift-curve slope and zero-lift drag coefficients from the referred studies before being deployed into the normal force model.

As described in Section “Measures of aerodynamic performance”, aerodynamic efficiency can also be quantified via the Glide Ratio ($GR=C_L/C_D$) and the Power Factor ($PF=C_L^{3/2}/C_D$) (see Fig. 14). The most striking difference between the behavior of $GR$ and $PF$ is the even spread of the various curves for $GR$ that cannot be seen for $PF$. For $4 \le Re \le 10$, the curves for $PF$ become more clustered together meaning that $PF$ becomes less dependent on $Re$ between this range. This finding is simply a consequence of the relationship between the curve fittings provided in Fig. 7, which mean that the ratio between $C_L^{3/2}$ and $C_{D}$ sees very little change when $Re$ ranges between 2 and 10 (see Fig. 14B). Although neither $GR$ nor $PF$ provide a means of comparing the efficiency of lift- and drag-based kinematics, being generic aerodynamic quantities, they do provide a means of comparing the aerodynamic efficiency of flight across various $Re$ (including those outside of the miniature insect domain). As such, in Fig. 14C, the aerodynamic efficiency of flapping flight in the miniature domain is compared to that at larger scales via the $PF$. In comparison to larger organisms, the aerodynamics of the miniature insects are remarkably inefficient, where the maximum $PF$ is approximately 76% lower than that of the *Drosophila*, and 90% lower than the *Bombus*. While quantification of $GR$ and $PF$ are indicators of the efficiency of *lift-based* flight, we have found from Fig. 12 that pure drag-based flight provides little-to-no improvement in efficiency when symmetric half-strokes are considered.

### Considering stroke asymmetry

The assessment of asymmetric half-stroke was carried out for $\theta _{amp} = 45^\circ$ (since that is the condition where lift- and drag-based kinematics coincide for *symmetric* half-strokes, and where the maximum effectiveness is achieved) and $f=$184 Hz. (which is simply the characteristic flapping frequency of the *P. placentis*, see Table 1). With regards to the control parameters $C_\phi$ and $C_\theta$, it is well-known that flapping waveforms characterized by a $C_{\phi }$ value close to 1 are somewhat unrealistic due to the large accelerations required to perform triangular flapping waveforms. In fact, the only feasible way to realize them is via revolving motion, rather than flapping. As such, real flapping waveforms are commonly sinusoidal or at least near-sinusoidal (Ellington 1984c; Dudley and Ellington 1990; Fry et al. 2005; Cheng and Sun 2018, 2021; Farisenkov et al. 2022), whereas, pitching waveforms can be a little more versatile (Elbatran et al. 2026). Hence, for this part of the assessment and without losing generality, $C_\phi$ will be kept at a value of 0, while $C_\theta$ will be varied between 0 and 5. Finally as described in Section “Motion representation”, $\sigma$ is varied across the range $-0.5 \le \sigma \le 0.5$ since this keeps us within the limits of the aerodynamic relations defined in Section “Quasi-steady aerodynamic framework”.

**Fig. 15:**
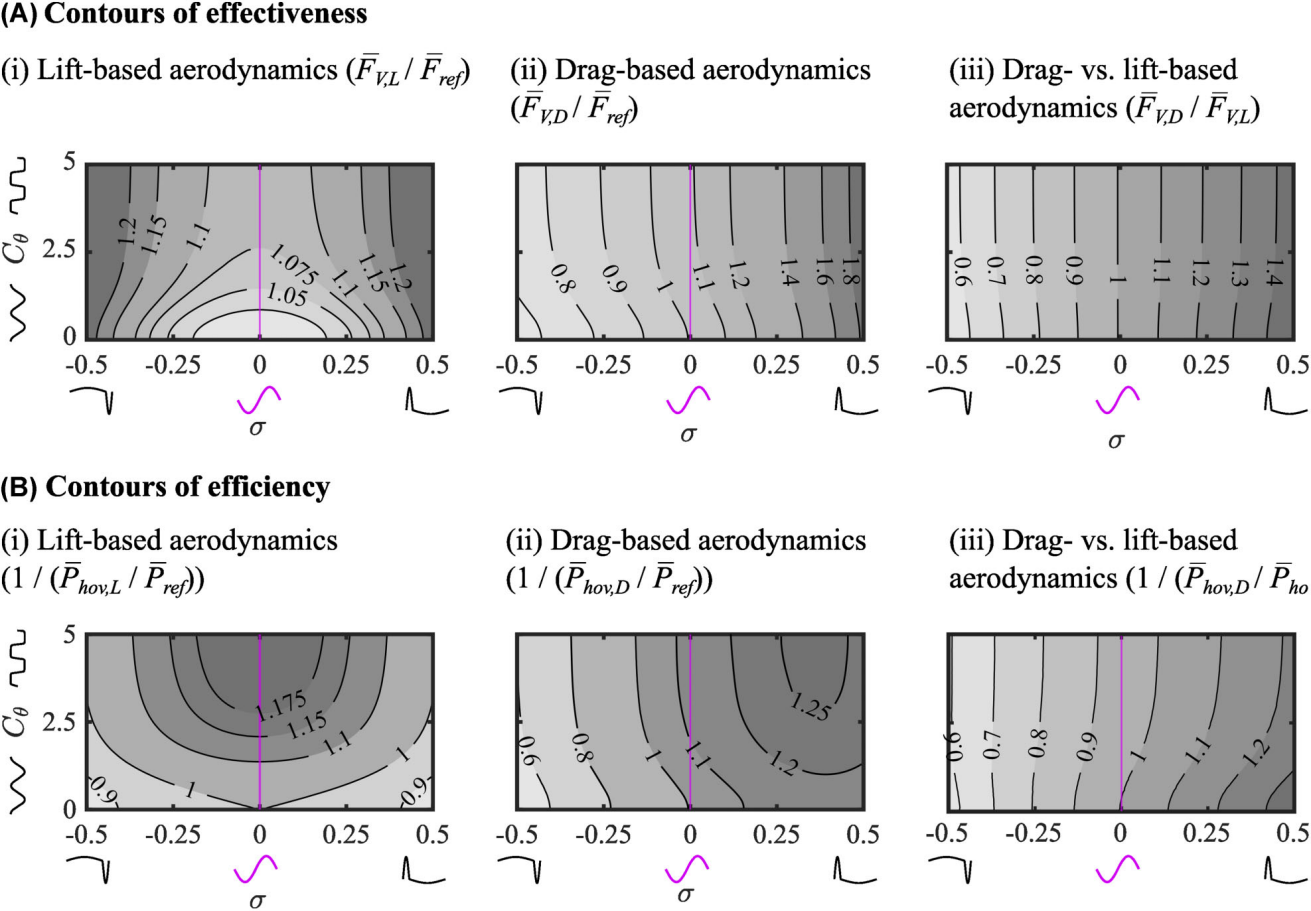
Stroke asymmetry results. Variations in (A) aerodynamic effectiveness (where $f=184$Hz) and (B) aerodynamic efficiency (i.e., $1/P_{hov}$) for $C_{\phi }=0$. In both (A) and (B), plots (i) and (ii) are associated with lift-based kinematics and drag-based kinematics, respectively, while (iii) provides a comparison between the two styles, via either the ratio between their aerodynamic effectiveness or their aerodynamic efficiency, for a particular $\sigma$ and $C_\theta$ combination. In (i) and (ii), effectiveness and efficiency have been normalized with respect to the values obtained for a symmetric stroke with a sinusoidal pitching variation (i.e., when $C_\theta =0$ and $\sigma =0$) and darker shades of gray indicate more desirable outputs. This demonstration is based on the numerical integration of Equations 12–16 with a $\theta _{amp}$ value of $45^\circ$ (i.e, the condition where lift- and drag-based kinematics coincide for symmetric half-strokes—see Fig. 11).

Figure 15A and B shows variations in effectiveness and aerodynamic efficiency, respectively, for lift- and drag-based kinematics, where the results are normalized with respect to the values for sinusoidal pitching angle variations and zero stroke-asymmetry (i.e, $C_{\theta }=0$ and $\sigma =0$). Generally speaking, for positive values of $\sigma$, effectiveness increases with stroke asymmetry for both lift- and drag-based kinematics and is at its greatest when $C_\theta =5$ (similar to the case of symmetrical half-strokes shown in Fig. 12A). On the other hand, for negative values of $\sigma$, as stroke asymmetry is decreased toward −0.5, we can see effectiveness increase for lift-based kinematics in the exact same fashion that it does for positive $\sigma$ values, while drag-based kinematics become increasingly less effective.

The fact that the sign of $\sigma$ (i.e., whether it is positive or negative) is irrelevant to the lift-based kinematics is somewhat unsurprising since the lift force is always counteracting the gravitational force regardless of whether we are considering the forward or back stroke. Whereas, the implication for the drag-based case is that effectiveness will increase while the down stroke is performed at a higher velocity than the upstroke—increasing the velocity of the upstroke relative to the downstroke has the opposite effect. This finding might seem somewhat trivial but, as explained in Section “Quasi-steady aerodynamic framework”, the exact nature of the aerodynamics in relation to positive or negative stroke-asymmetry is in fact non-obvious owing to the significant increase in force coefficients as *V* is reduced toward 0. Additionally, it was also outlined in Section “Introduction” that the exact manner in which miniature insects carry out stroke asymmetry (i.e., whether or not they use a quicker downstroke or upstroke) has been subject to conflicting results.

The rate at which effectiveness increases with $\sigma$ is much greater for drag-based kinematics than for lift-based kinematics—hence, drag-based kinematics become increasingly more effective than lift-based kinematics with increasing $\sigma$ [see Fig. 15A(iii)]. Then, with regards to aerodynamic efficiency, while aerodynamic efficiency decreases for lift-based kinematics as $\sigma$ becomes more positive or negative, efficiency decreases as $\sigma$ becomes more negative for drag-based kinematics but actually increases as $\sigma$ becomes more positive, up to a value of approximately $\sigma \approx 0.4$—beyond $\sigma \approx 0.4$ efficiency begins to decrease. Overall, the above findings imply that a purely drag-based stroke, who’s half-strokes are asymmetric in the range $0 \le \sigma \le 0.4$, is both more effective and aerodynamically efficient at miniature scales than both a lift-based stroke with the same degree of stroke asymmetry, and a lift- or drag-based stroke that have zero stroke asymmetry.

**Fig. 16:**
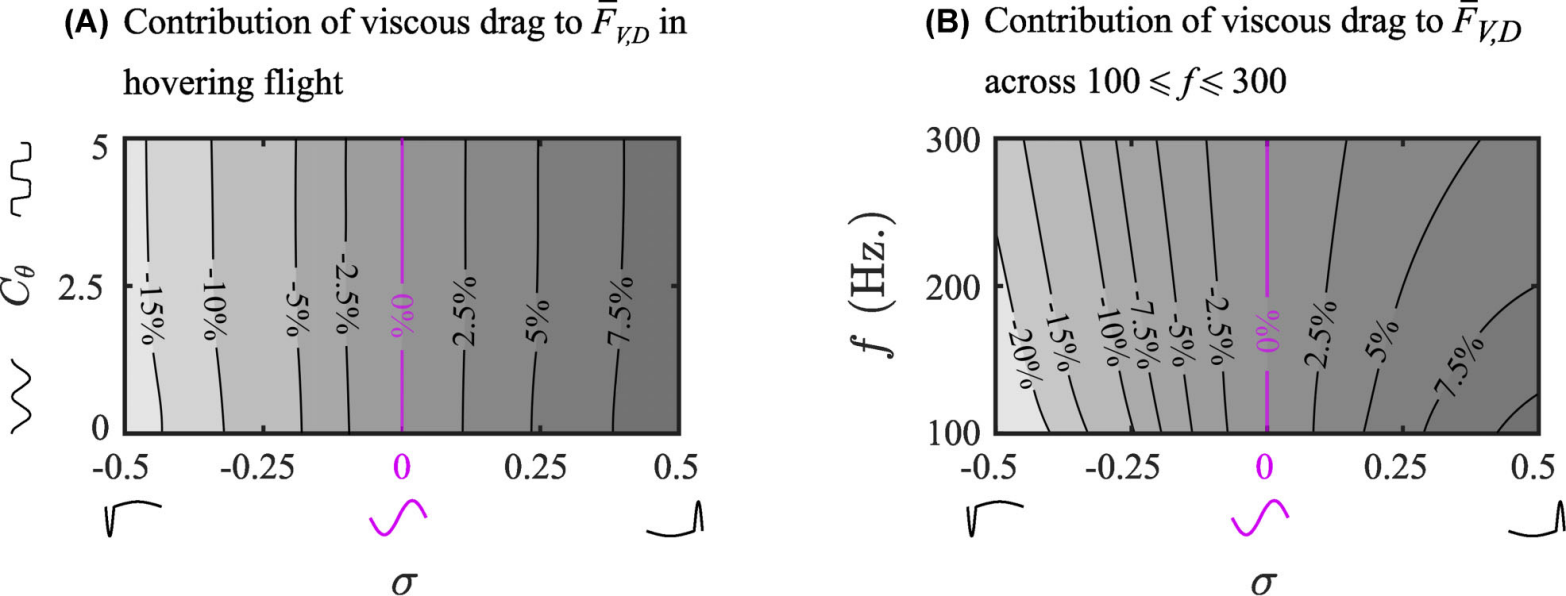
Contours of the percentage contribution of the skin friction drag component to $\bar{F}_{V,D}$ across a single stroke for hovering flight according to $\sigma$ values ranging $-0.5 \le \sigma \le 0.5$ and (A) $C_\theta$ values ranging 0–5 and (B) *f* values ranging 100 Hz $\le f \le$ 300 Hz, where $\theta _{amp}=45^\circ$. In both plots $C_\phi =0$, while in (B) $C_\theta =5$.

Probably the most curious finding from those points discussed above is that, with increasing $\sigma$, aerodynamic efficiency either decreases or increases depending on whether the stroke is lift- or drag-based, respectively. To explain this, we turn our attention toward the key feature of miniature-scale aerodynamics that makes it such a distinct domain—that being the heightened presence of viscous flow effects (i.e., skin friction drag). In Fig. 7B, we saw that the zero-lift drag coefficient is considerably large for $Re$ values within the miniature domain. Hence, it is instructive to consider the net contribution of the skin friction component toward $\bar{F}_{V,D}$ in the drag-based profile, and how this contribution is altered for various degrees of stroke asymmetry. Note that skin friction drag is calculated by isolating the $C_{D,0}$ component of Equation 10 and deploying it in to Equation 13 for a particular kinematic waveform configuration. Therefore, for a pure lift-based stroke, the skin friction component cannot be utilized for augmenting the lift force (and therefore $F_{V,L}$) at all, since Equation 10 of the normal force model does not feature in Equation 12. Figure 16A shows contours of the net time-averaged viscous drag across a stroke as a percentage of $\bar{F}_{V,D}$ in hovering flight for $\theta _{\mathrm{amp}}=45^\circ$, *f*=184 Hz, and $C_\phi = 0$, through the ranges $-0.5 \le \sigma \le 0.5$ and $0 \le C_{\theta } \le 5$. The amount by which skin friction contributes to the overall vertical force increases from 0% to almost 10% as $\sigma$ is increased toward 0.5, whereas its contribution reaches as large as −15% as $\sigma$ is decreased toward $-0.5$ (i.e., the viscous contribution is in the *downward* direction for negative values of $\sigma$).

Figure 16B shows how the skin friction contribution varies across a full stroke as according to $\sigma$ and *f* while $C_\phi$ and $C_\theta$ are 0 and 5, respectively. The contours illustrate how the tendency for viscous drag to have a larger contribution to the overall vertical force with increasing $\sigma$ is largely dependant on *f*. To explain why this is the case, the reader is referred back to Fig. 9A which shows how $Re_{\mathrm{max}}$ increases with both $\sigma$ and *f*. Hence, it appears that in order to take maximum advantage of the viscous environment we must first be operating at miniature-scale $Re$ (where one way to achieve this by reducing *f*) and then increase $\sigma$ as far as possible without exiting from the miniature-scale $Re$ domain. In other words, the results suggest that, at miniature-scale $Re$, a positively asymmetric drag-based stroke can take a notable advantage of air viscosity in a way that is not possible for a lift-based stroke at miniature scale $Re$ or any stroke characterized by $Re$ values larger than that of miniature scales. This finding pinpoints a potential precise reason why we might see a large dependency on drag in the miniature domain that we see nearly nowhere else in flapping wing flight.

### Assessing the implications of using *Re*-dependant aerodynamic coefficients

The aerodynamic characteristics of miniature insect wings are highly sensitive to changes in the Reynolds number. In previous quasi-steady studies [e.g., Berman and Wang (2007) and Nabawy and Crowther (2015)], the wing’s lift and drag characteristics have been assumed to be dependant on the wing’s geometry and geometric angle of attack only. However, as can be seen in Fig. 7, in the miniature domain, the wing’s lift-curve slope and zero lift drag coefficient vary significantly over a relatively small $Re$ range (i.e., $0 < Re < 40$). Hence, the wing’s aerodynamic coefficients will vary continuously throughout a wing stroke. The above discussion implies that for a given wing morphology, the wing’s lift and drag coefficient at any point during the stroke will significantly depend not only on the wing’s angle of attack but also on the wing’s characteristic $Re$—this has been the approach taken for producing all of the results presented in this paper thus far. However, in this section, we wish to reveal any implications which arise from modeling the aerodynamics in this manner, via comparison to results for which $C_{L, \alpha }$ and $C_{D,0}$ have been assumed to be fixed throughout the wing stroke [i.e., the approach which is sufficient for analysis of larger insects (Lentink and Dickinson 2009)].

**Fig. 17:**
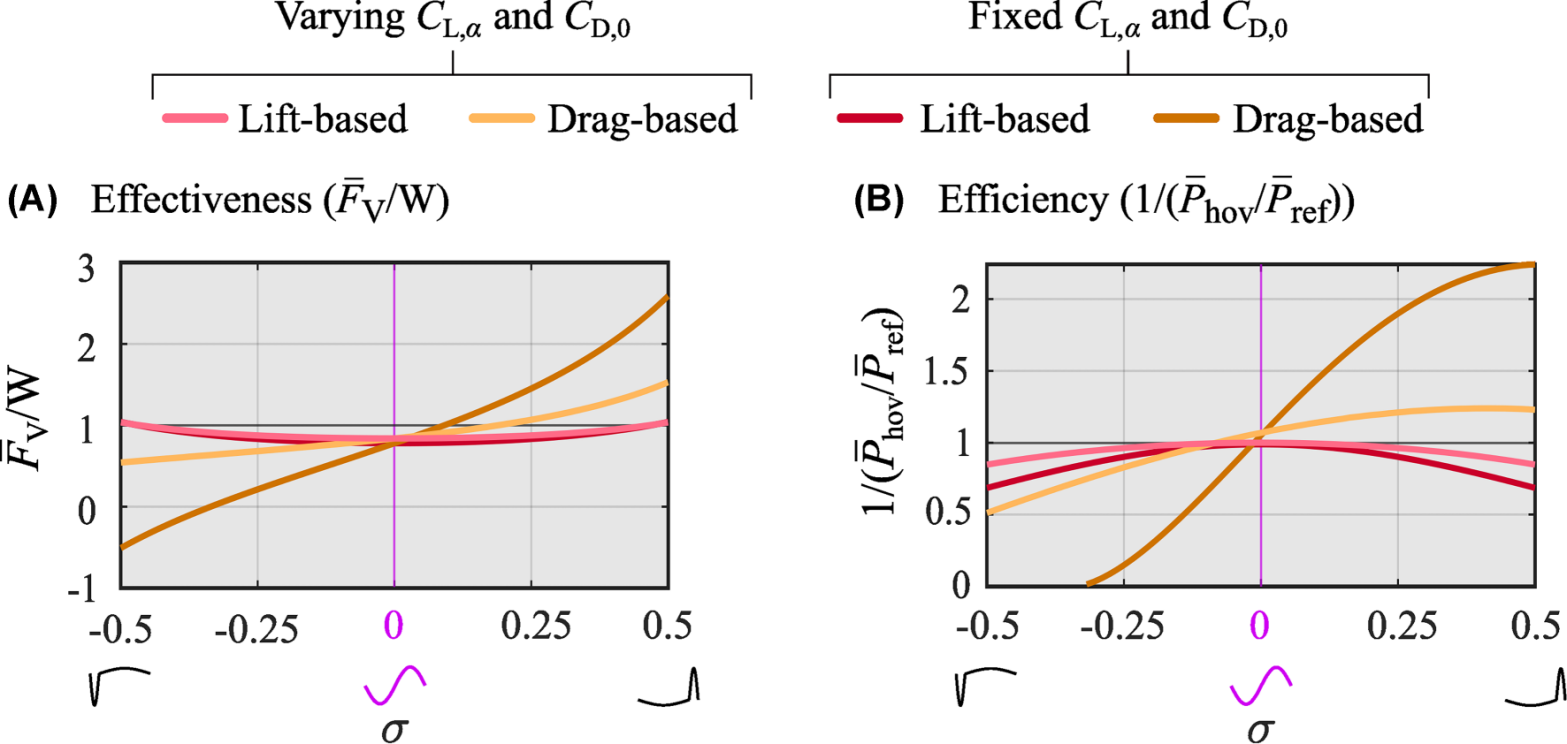
(A) Effectiveness and (B) aerodynamic efficiency for sinusoidal *V* and $\alpha$ waveforms for lift- (red curves) and drag-based (orange curves) kinematics for $\sigma$ values across the range $-0.5 \le \sigma \le 0.5$. Lighter shaded red and orange curves illustrate the results for when the normal force model is parameterized by $C_{L,\alpha }$ and $C_{D,0}$ values which vary as according to the wing’s instantaneous $Re$. Whereas, darker shaded red and orange curves illustrate the results for when $C_{L,\alpha }$ and $C_{D,0}$ are fixed corresponding to $Re=10$ only (where $Re=10$ was chosen since this is the value typically used to characterize flight in the miniature domain). $\bar{F}_{\mathrm{V}}$ has been normalized with respect to insect weight, *W*, whereas $\bar{P}_{\mathrm{hov}}$ has been normalized with respect to $\bar{P}_{\mathrm{ref}}$ which is the $\bar{P}_{\mathrm{hov}}$ value for a symmetric lift-based stroke when using varying $C_{L,\alpha }$ and $C_{D,0}$ values (i.e., that of the dark red curve at $\sigma =0$).

Figure 17 shows the effectiveness and aerodynamic efficiency of both lift- (red curves) and drag-based (orange curves) kinematics for sinusoidal waveform variations (i.e., $C_\phi = C_{\theta } = 0$) across the range $-0.5 \le \sigma \le 0.5$. In order to demonstrate the effects of parameterizing the aerodynamic model using $Re$-dependant aerodynamic coefficients, the figure compares the effectiveness and efficiency values calculated when the normal force model is parameterized via two methods. The first method, which is that used in all of the preceding analyses in this paper, parameterizes the normal force model via $C_{L,\alpha }$ and $C_{D,0}$ values which vary as according to the wing’s instantaneous $Re$ (illustrated via lighter shaded red and orange curves). The second method parameterizes the normal force model via $C_{L,\alpha }$ and $C_{D,0}$ values which are fixed and correspond to $Re=10$—that is, which is typically used to characterize flight in the miniature domain (illustrated via darker shaded red and orange curves). Only the results for sinusoidal *V* and $\alpha$ variations have been shown here in order to avoid over-convoluting the discussion and distracting from the key conclusions. With that being said, it should be noted that the overall trends illustrated in Fig. 17 can be considered generic and similar conclusions are drawn for all other $C_{\phi }$ and $C_{\theta }$ parameter value couplings. The key take-away from the results shown in Fig. 17 is that both effectiveness and aerodynamic efficiency are more sensitive to changes in $\sigma$ when parameterizing the normal force model via the second method (i.e., when using $C_{L,\alpha }$ and $C_{D,0}$ values which are fixed and correspond to $Re=10$), particularly for drag-based kinematics. In other words, the two performance metrics change more significantly as $\sigma$ is made more positive or more negative when we do not consider aerodynamic coefficients that are $Re$-dependant. The described effect is so pronounced for drag-based kinematics that hovering flight actually becomes unachievable at $\sigma \approx -0.3$. On the other hand, the effect is less significant when considering lift-based kinematics.

The reason why the phenomenon outlined above arises can be explained via the following discussion in which we consider a drag-based stroke that is positively asymmetric. For drag-based aerodynamics, when $\sigma$ increases positively, the wing’s overall translational velocity on the downstroke is increased corresponding to larger values of $Re$. Whereas conversely, on the upstroke, as $\sigma$ becomes more positive, the overall translational velocity becomes smaller and, therefore, so do the corresponding values of $Re$. Considering this, and referring to Fig. 7, we can see that larger and smaller values of $Re$ correspond to smaller and larger values of the aerodynamic coefficients, respectively. As such, although positively increasing $\sigma$ corresponds to an increase in the overall effectiveness and aerodynamic efficiency for drag-based kinematics, considering $Re$-dependant values of the aerodynamic coefficients dampens this effect by decreasing and increasing the aerodynamic coefficients on the downstroke and the upstroke, respectively. The reverse is true as $\sigma$ becomes more negative, whereby the aerodynamic coefficients are increased and decreased on the downstroke and upstroke, respectively, rendering the same dampening effect on effectiveness and aerodynamic efficiency which become smaller at a lower rate than when using fixed $C_{L,\alpha }$ and $C_{D,0}$ values. Although the above discussion is directed toward drag-based kinematics, the overarching conclusions are generic and also apply to lift-based kinematics. However, as can be seen in Fig. 7, the described dampening effect is more subtle for lift-based kinematics.

Applied to the real world, the above findings suggest that the high sensitivity of a miniature insect’s aerodynamic characteristics to changes in $Re$ may be disadvantageous to the insect’s aerodynamic performance when considering changes in stroke-asymmetry. If the aerodynamic coefficients which characterize a miniature insect wing hardly varied throughout the wing stroke, then we might see a more significant improvement in effectiveness and aerodynamic efficiency as $\sigma$ becomes more positive (as shown by the darker red and orange curves). Conversely, however, for negative values of $\sigma$, it is also true that aerodynamic efficiency is much improved for a drag-based stroke when $Re$-dependant aerodynamic characteristics are considered, since efficiency falls at a slower rate as $\sigma$ becomes more negative. Therefore, whether or not it is an advantage to have highly $Re$-dependant aerodynamic characteristics is dependant on whether stroke asymmetry is negative or positive. Overall, our results suggest that, when considering a drag-based stroke, it is more beneficial for a miniature insect to use positive stroke asymmetry, which implies that their highly $Re$-dependant aerodynamic characteristics are somewhat of a hindrance to their performance. However the overarching conclusion is that the high sensitivity of a miniature insect wing’s aerodynamic characteristics to changes in $Re$ dampens any change in performance as $\sigma$ is made more positive or negative.

## Discussion and concluding remarks

The reason why miniature insects utilize swimming-like flapping profiles has been alluded toward in previous works mostly through intuition and speculation. In Section “Introduction”, the few studies which have approached the problem directly were discussed, with some investigations finding a clear aerodynamic advantage to using drag-based profiles at $Re=10.6$ (Cheng and Sun 2018) and some others finding no advantage at all across a range of $Re$ relevant to the miniature domain (Jones et al. 2015; Engels et al. 2021; Shen et al. 2022). Considering the results provided in the present study, it makes sense now why Jones et al. (2015) and Shen et al. (2022) found that a pure lift-based stroke is slightly more effective than a pure drag-based stroke at miniature scales, since they drew these conclusions based on strokes that were symmetric about the half period. As illustrated in Section “Aerodynamic performance for symmetric profiles”, for symmetric half-strokes, a pure drag-based profile will never be more effective than a lift-based profile and it is only when the pitching amplitude was defined as $45^\circ$ when the two cases coincide—in all other cases, the lift-based stroke was more effective than the drag-based stroke. Additionally, for the most part, lift-based kinematics are slightly more aerodynamically efficient when utilizing symmetric half-strokes with the exception of when the flapping profile becomes triangular, which is known to be an unrealistic expectation of flapping wings in the real world.

Interestingly, after assessing the symmetric case, Jones et al. (2015) introduced stroke asymmetry and found that a slight augmentation in the vertical force could be achieved for the drag-based kinematics. However, no comparison to asymmetric lift-based kinematics was provided meaning that no meaningful conclusions regarding the advantages of asymmetric drag-based kinematics could be drawn. In this study, we considered stroke asymmetry for both lift- and drag-based kinematics by varying the degree to which the period of one half-stroke is larger/smaller than the subsequent half-stoke, via the parameter $\sigma$. Interestingly, we find that introducing asymmetry (for both positive and negative values of $\sigma$) does have its advantages with lift-based kinematics with regards to effectiveness, but renders the flight style slightly less aerodynamically efficient. On the other hand, increasing asymmetry for the drag-based stroke (by making $\sigma$ more positive, only) leads to significant augmentation of both effectiveness and aerodynamic efficiency at miniature scales. To explain this, in Section “Considering stroke asymmetry”, we have isolated the effects of flow viscosity via the $C_{D,0}$ component of the normal force model—which is arguably the key feature that distinguishes the domain of miniature-scale aerodynamics from any other flight category. We found that, as $\sigma$ is increased above 0 for both a lift- or drag-based stroke, a force imbalance arises that is largely owing to the skin friction component. This force imbalance can only be utilized for weight support using drag-based kinematics and, therefore, we suggest that drag-based kinematics do have a specific benefit at miniature scales in order to take advantage of viscous flow effects, so long as asymmetric half-strokes are employed. Earlier in this paper, we also mentioned how there were mixed-findings with regards to whether miniature insects perform slower upstrokes and quicker downstroke or vice-versa. As per our results, we suggest that positive stroke asymmetry (i.e., a faster downstroke) will bring aerodynamic benefits for a pure-drag-based stroke while negative stroke asymmetry negatively effects the aerodynamics. On the other-hand, it makes no difference whether stroke asymmetry is positive or negative for lift-based kinematics since the effects are symmetric about $\sigma =0$. Since real miniature insects perform more complex wing kinematics that are somewhat of a hybrid between pure lift- and drag-based kinematics, it is hard to make any firm conclusions about the most sensible way for miniature insects to flap their wings. However, what we have shown is that by performing positive stroke asymmetry, it is possible to take advantage of a highly viscous environment for a stroke that depends on drag for the upwards vertical force vector, which is something that is certainly pertinent to miniature insects.

The sensitivity of miniature-scale aerodynamic wing characteristics to changes in $Re$ is unprecedented and must also be highlighted. Outside of the miniature domain the aerodynamic characteristics of insect wings can be considered to stay relatively constant across $Re$ ranges spanning several orders of magnitude (Lentink and Dickinson 2009). Whereas, the reality of miniature-scale aerodynamics is starkly different and the $C_{L,\alpha }$ and $C_{D,0}$ values of the *P. placentis* wing increase by a multiple 2 and 3, respectively, as $Re$ falls only slightly from 10 to 2. Moreover, the very unique aerodynamic environment faced by miniature insects is arguably what makes flight achievable in some instances. For instance, Fig. 17B shows how hovering flight cannot be achieved for certain values of $\sigma$ when $C_{L,\alpha }$ and $C_{D,0}$ are kept fixed throughout the stroke; however, when those characteristics become $Re$-dependant (as according to the expressions in Fig. 7), the effects of stroke-asymmetry are dampened and hovering flight becomes possible. Of course, those instances (when hovering flight is not possible) occur for negative values of $\sigma$ which we have shown create undesirable aerodynamic performance characteristics in hovering flight—however, we simply wish to emphasize the significant influence that the flow characteristics have over such a small $Re$ range. Hence, in future endeavors, we strongly suggest that a particular focus must be placed on further revealing the influence of the characteristics shown in Fig. 7, if we are to fully understand flight in the miniature-scale domain.

With regards to the limitations of the present study, primarily, it must be remembered that the swimming-like flapping profiles performed by miniature insects are somewhat of a hybrid between the two extreme flapping profiles considered, in which both lift and drag vectors are used significantly to counteracting gravity. As such, a more extensive study in which the various kinematic profiles that exist between the two considered extreme cases is necessary for more concrete real-world conclusions. However, the purpose of the present study was not to model the exact kinematics performed by miniature insects but rather to provide some initial clarity regarding the disparity (or lack there of) between pure lift- and drag-based flight. Second, additional aerodynamic mechanisms that are relevant to flapping wing flight (i.e., wake-capture, wing rotation, and the added-mass effect) were not considered in this paper. However, translational effects (which are the dominant aerodynamic effect for flying insects) were all that were necessary to model the topology of the effectiveness and aerodynamic efficiency of pure lift- an drag-based flapping styles. As outlined in Section “Modeling assumptions”, the effects of wake capture can be safely neglected owing to the augmented energy dissipation of rotational flows, owing to enhanced viscous effects at miniature scales. Furthermore, the net effects of wing rotation and added mass are negligible for a stroke that has no advance/delay in pitching with respect to the translational motion. We also assume the system to be perfectly elastic meaning that only aerodynamic power is considered (Phan and Park 2018). Finally, it must also be pointed out that the lack of existing relevant data makes an in-depth validation analysis of the present quasi-steady model not possible. With that being said, we have carried out a validation in Section “Model verification” using the limited data that is available but we emphasize that the field is in relative infancy at present, particularly with regards to the quasi-steady analysis. Hence, there exists a large gap for further data before the quasi-steady approach can be conveniently validated in the miniature domain.

## Funding

None declared.

## Data Availability

All data are contained within the article.
